# Refining Ligand Poses in RNA/Ligand Complexes of Pharmaceutical Relevance: A Perspective by QM/MM Simulations and NMR Measurements

**DOI:** 10.1021/acs.jpclett.4c03456

**Published:** 2025-02-10

**Authors:** Gia Linh Hoang, Manuel Röck, Aldo Tancredi, Thomas Magauer, Davide Mandelli, Jörg B. Schulz, Sybille Krauss, Giulia Rossetti, Martin Tollinger, Paolo Carloni

**Affiliations:** † JARA-Brain Institute Molecular Neuroscience and Neuroimaging (INM-11), Forschungszentrum Jülich, 52425 Jülich, and RWTH Aachen University, 52056 Aachen, Germany; ‡ Institute of Organic Chemistry and Center for Molecular Biosciences Innsbruck (CMBI), University of Innsbruck, Innrain 80/82, 6020 Innsbruck, Austria; § Institute for Neuroscience and Medicine (INM-9), Forschungszentrum Jülich, 52425 Jülich, Germany; ∥ Department of Neurology, Medical Faculty, RWTH Aachen University, 52074 Aachen, Germany; ⊥ Institute of Biology, University of Siegen, 57076 Siegen, Germany; # Jülich Supercomputing Center (JSC), Forschungszentrum Jülich, 52425 Jülich, Germany

## Abstract

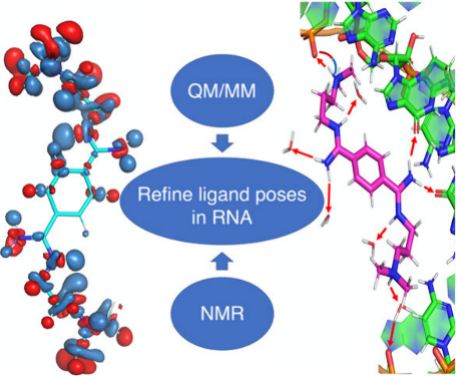

Predicting the binding poses of ligands targeting RNAs is challenging. Here, we propose that using first-principles quantum mechanics/molecular mechanics (QM/MM) simulations, which incorporate automatically polarization effects, can help refine the structural determinants of ligand/RNA complexes in aqueous solution. In fact, recent advances in massively parallel computer architectures (such as exascale machines), combined with the power of machine learning, are greatly expanding the domain of applicability of these types of notoriously expensive simulations. We corroborate this proposal by carrying out a QM/MM-based study on a ligand targeting CAG repeat-RNA, involved in Huntington’s disease. The calculations indeed show a clear improvement in the ligand binding properties, and they are consistent with the NMR measurements, also performed here. Thus, this type of approach may be useful for practical applications in the design of ligands targeting RNA in the near future.

Because the majority of the human genome is transcribed into RNA, but only a small fraction encode proteins, the therapeutic focus is increasingly shifting to RNAs. Noncoding RNAs, such as microRNAs, are critical regulators of gene expression, and their dysregulation is associated with a number of diseases.^1−3^ Thus, targeting noncoding RNAs may open new avenues for therapeutic development, particularly where mutated or aberrant RNA expression is implicated in diseases, as highlighted by the recent Nobel Prize for microRNA research. Equally important is the targeting of mRNAs, which can modulate the expression of proteins that are otherwise difficult to target directly with traditional drug discovery approaches. These include proteins that form fibrils in neurodegenerative diseases, which lack accessible binding sites for traditional small molecules.^4^ An example from our team here is the identification of a small molecule—furamidine—that can affect the biosynthesis of the huntingtin protein involved in Huntington’s disease by binding to the pathogenic repeat CAG expansion sequences^5^ (see Section 1 in the Supporting Information for details).

Non-covalent small molecules (ligands) targeting RNAs have certain inherent advantages over larger therapeutic approaches (such as antisense therapies) such as better cellular uptake, ease of chemical optimization, and more controlled pharmacokinetics. However, this approach faces significant obstacles, including a lack of chemical diversity, low selectivity, and high dosage requirements. These limitations underscore the need for the rational design and optimization of RNA-targeting small molecules to expand the spectrum of therapies. Because RNAs (i) typically lack obvious ligandable pockets and (ii) exhibit great flexibility, methods that provide structural dynamics information are particularly valuable. Machine learning (ML) approaches have made impressive progress in predicting protein structure, and AlphaFold 3 shows great potential for RNA. However, the predictive power is currently limited by the paucity of experimental information, from cryo-EM, X-ray crystallography or nuclear magnetic resonance (NMR) (see https://hariboss.pasteur.cloud/). Importantly, NMR has the bonus of providing dynamic information about the adducts in solution, which may be very important for the design of ligands targeting these highly plastic molecules. Unfortunately, NMR is not without limitations. Experimental NOE data are incorporated into the structure refinement as inter- and intramolecular distance constraints, the boundaries of which are derived from relative NOE intensities in a somewhat subjective manner. In addition, due to the relatively low density of nonexchangeable hydrogen atoms in RNA, fewer intermolecular distance restraints are available for structure refinement compared to protein–ligand complexes. To overcome this limitation, longer mixing times can be used in NOESY experiments to increase the range and number of observable intermolecular NOE contacts. However, this approach can lead to spin diffusion, which introduces ambiguities in experimentally derived distance restraints due to indirect magnetization transfer by nearby protons.^6,7^ Additional ambiguities in the distance constraints used for structure refinement may arise from the limited chemical shift dispersion of RNA signals.^8^ Taken together, these experimental challenges and drawbacks can lead to uncertainties in the precise binding mode of the ligand molecules based on NOEs.

Complementary to NMR, molecular dynamics (MD) simulations can, in principle, be of great help in providing information on the dynamics, especially regarding the mobility of the ligands. Unfortunately, MD may also have difficulties in accurately describing RNA–drug interactions.^9^ This may be due, at least in part, to the difficulty of force fields to account for large polarization effects on the (usually positively charged) ligands by the highly negatively charged RNAs. Such effects can be further enhanced by the absence of a buried binding pocket, which leaves the ligand exposed to the solvent. Two strategies can be considered to address this issue. Accurate polarizable force field-based MD provides the ability to modify the electrons distribution within biomolecules, enabling more accurate replication of interactions and dynamics of RNA^10^ with promising results, although limitations do remain^11^ (see section 2 in Supporting Information). Alternatively, first-principles quantum mechanics/molecular mechanics (QM/MM) MD allows the explicit description of the electron degrees of freedom, including polarization effects, without the need of any ad-hoc parametrization. QM/MM simulations are well-established tools for elucidating non-covalent small molecule interactions with proteins,^12^ including structural refinement of protein/ligand complexes.^13^ However, the scenario is dramatically different for RNA/ligand complexes, which might be more difficult to model by biomolecular force fields than proteins: to the best of our knowledge, QM/MM MD simulations of RNA/non-covalent ligand complexes have never been attempted. QM/MM has shown instead to help rationalize problems seen in MD simulations of protein-RNA complexes.^200,201^ This approach seems very timely, as (i) it may cover sub-nanosecond dynamics on modern supercomputers^14^ thanks to current computational architectures in the exascale era,^15,16^ combined with the power of parallel computing;^17^ (ii) it is becoming more and more important as a data source for training. Here, we explore the possibility of using massively parallel QM/MM MD validated by raw data (such as NMR chemical shifts) as a refinement tool for NMR structures of RNA/small molecules complexes.

As a practical application of these techniques, we focus on an RNA homoduplex of sequence GCAGCAGCUUCGGCAGCAGC (CAG RNA hereafter), in complex with the symmetric DB213 ligand, for which an NMR ensemble of five structures based on NOEs constraints has been published (Figure 1 and Chart 1).^18^ This complex is of interest in the context of Huntington’s disease pharmacology (See section 1 in Supporting Information). Here, the ligand binds to the major groove of the CAG RNA homoduplex, forming a hairpin structure. Comparison is also made with NMR chemical shifts measured here on the complex and on the ligand in water. For our QM/MM MD calculations, we use the MiMiC interface.^19,20^ MiMiC (coded by a consortium of research groups including ours) is flexible software that uses a multiple-program, multiple-data model with loosely coupled programs, achieving fast data exchange between programs through a lightweight MPI-based communication library. MiMiC implements an efficient electrostatic embedding QM/MM MD protocol combining the plane wave density functional theory (DFT) CPMD code^21^ and the GROMACS program.^22^

**Figure 1:**
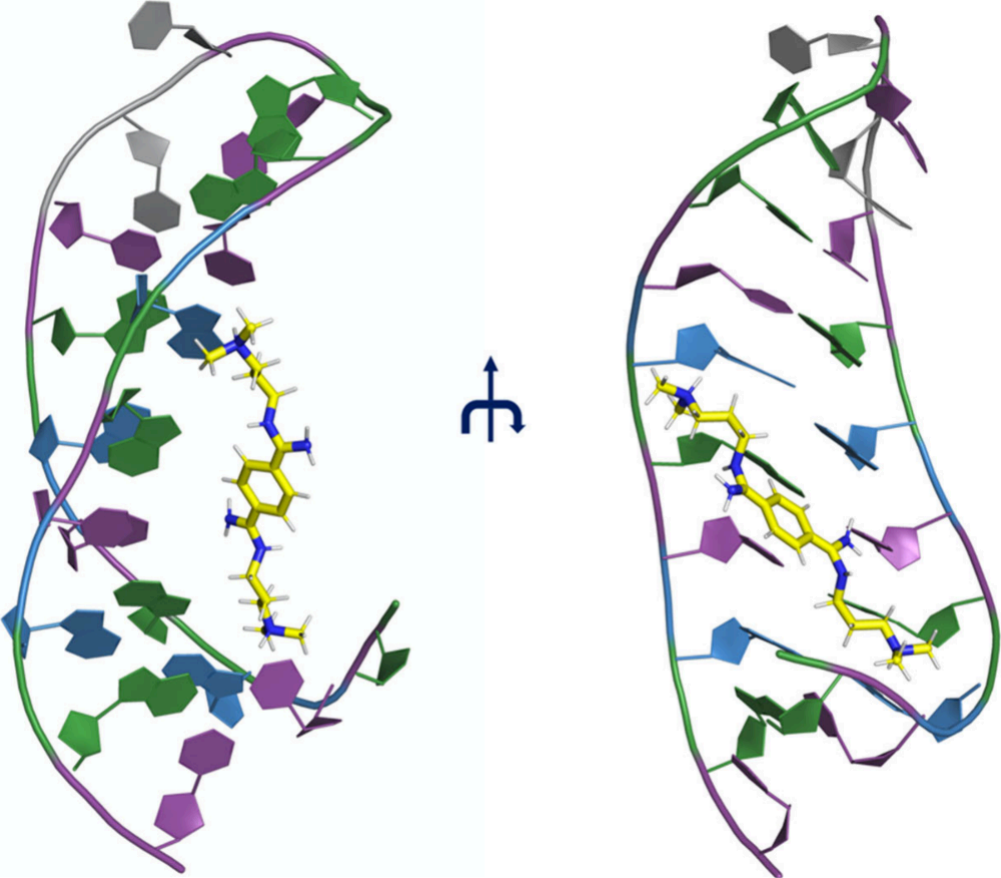
NMR structure of CAG RNA in complex with the DB213 ligand.^18^ The ligand is in yellow stick representation, while the CAG RNA is in cartoon representation with the C, A, G, and U nucleobases in violet, blue, green, and gray, respectively.

**Chart 1:**
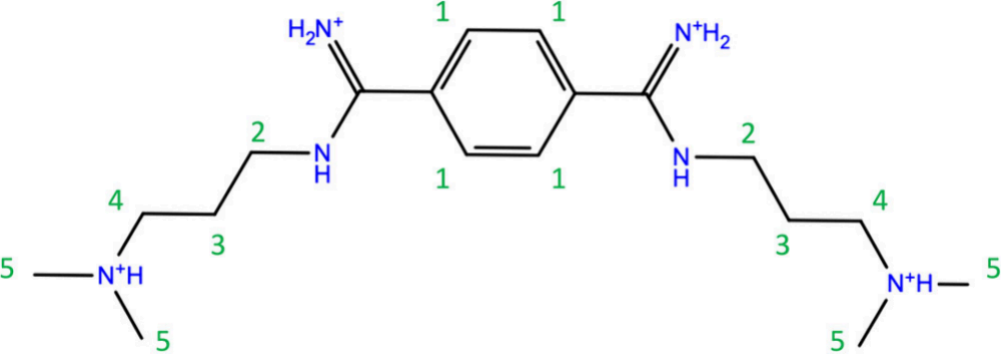
Ligand DB213

After a preparation phase by MD in explicit solvent, in which the complex was restrained to its NMR structure, we performed 0.1 ns QM/MM MD simulation, in which the ligand was treated at the DFT level (section 3.1 in the Supporting Information). The calculations were based on the first deposited NMR structure of the ligand/CAG RNA complex.^18^ The RMSD values of the backbone atoms relative to the initial NMR structure oscillate around 0.15 nm (Figure 2a), a value that is only slightly larger than the RMSDs among the five NMR structures (from 0.08 to 0.11 nm). The regions that exhibit the largest fluctuations are, as expected, those at the termini and the loop. The region near the ligand’s binding pose (A3, G4, C5, A15, G16, C17) is relatively less flexible (Figure 2b).

**Figure 2:**
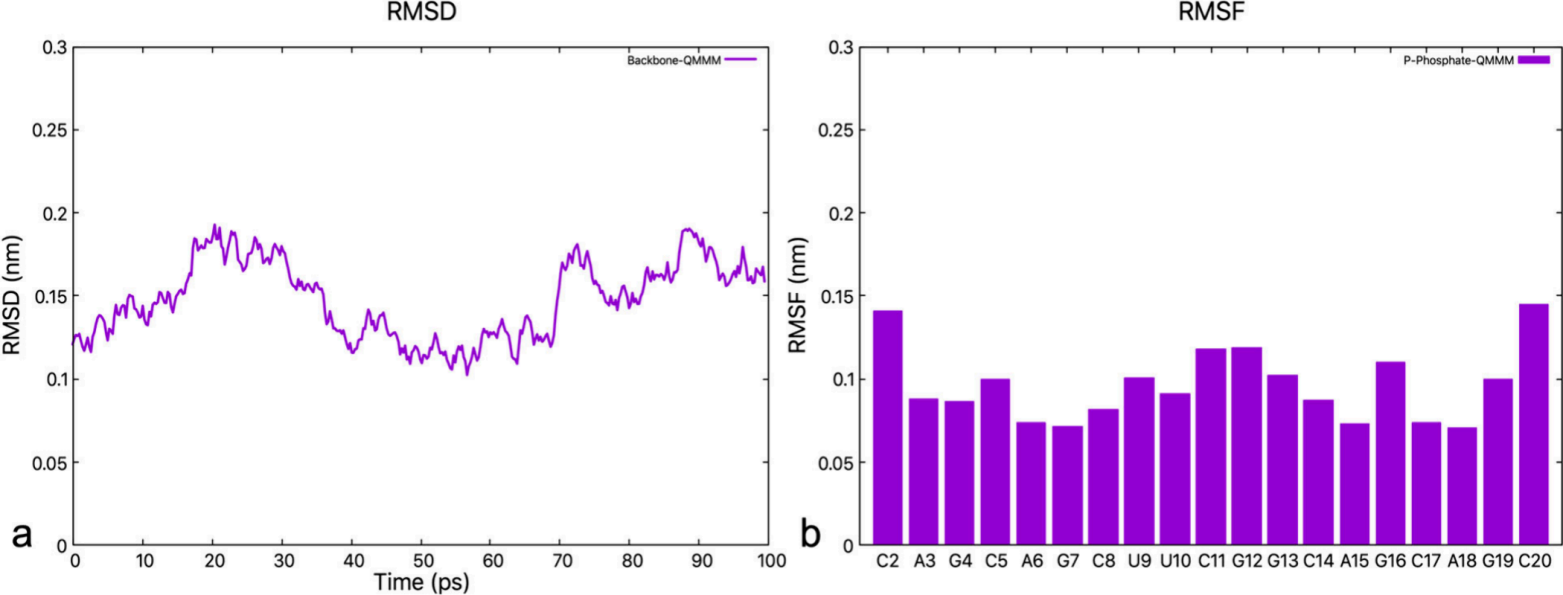
(a) RMSD of the CAG RNA backbone as a function of the simulated time. (b) RMSF histogram of the phosphorus atom in the phosphate group of each nucleobase.

The ligand rearranges significantly during the QM/MM dynamics, as shown by its RMSD. This is calculated by keeping the RNA backbone fitted to the NMR structure: it increases to ∼0.3 nm during the first 40 ps and then fluctuates around 0.25 nm for most of the rest of the dynamics (Figure 3). We therefore analyzed the last 60 ps.

**Figure 3:**
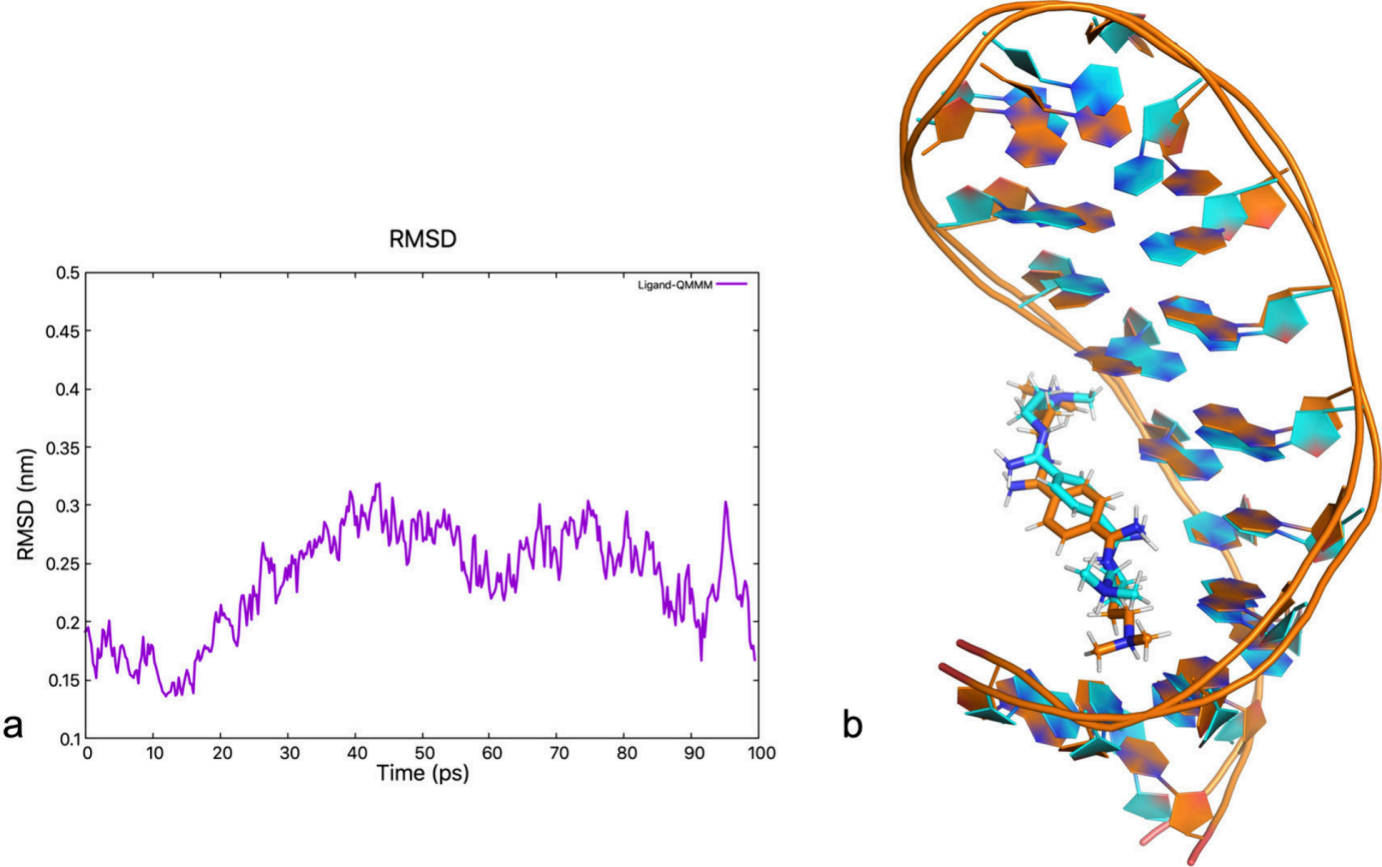
QM/MM simulation (a) RMSD of the DB213 ligand relative to the NMR structure as a function of simulated time. The RNA backbone is fitted to the NMR every time step. (b) Superimposition of the QM/MM structure after 100 ps (blue) with the NMR 1 structure (orange, RMSD = 0.17 nm). Those with the other four NMR structures are shown in Figure S1.

In the QM/MM MD, the ligand forms more H-bonds than in the NMR 1 structure (Figure 4a): the H5 and H7 hydrogen atoms form hydrogen bonds with O6@G4 and O6@G16, and H29 forms a hydrogen-bonded salt bridge with OP2 in the phosphate group of A15 (Figure 4b). These interactions are absent in the NMR model 1 structure. These interactions are mostly kept during dynamics (Table S1). The other H-bond functionalities of the ligand are exposed to solvent and form hydrogen bonds with water molecules. Hydrophobic interactions are formed between the ligand and C2, A3, G4, C14, A15, and G16 of CAG RNA, as in the case of the NMR structure. A more detailed discussion on the ligand/RNA interactions in NMR model 1 as well as in the other 4 NMR structures is offered in section 4 in Supporting Information.

**Figure 4:**
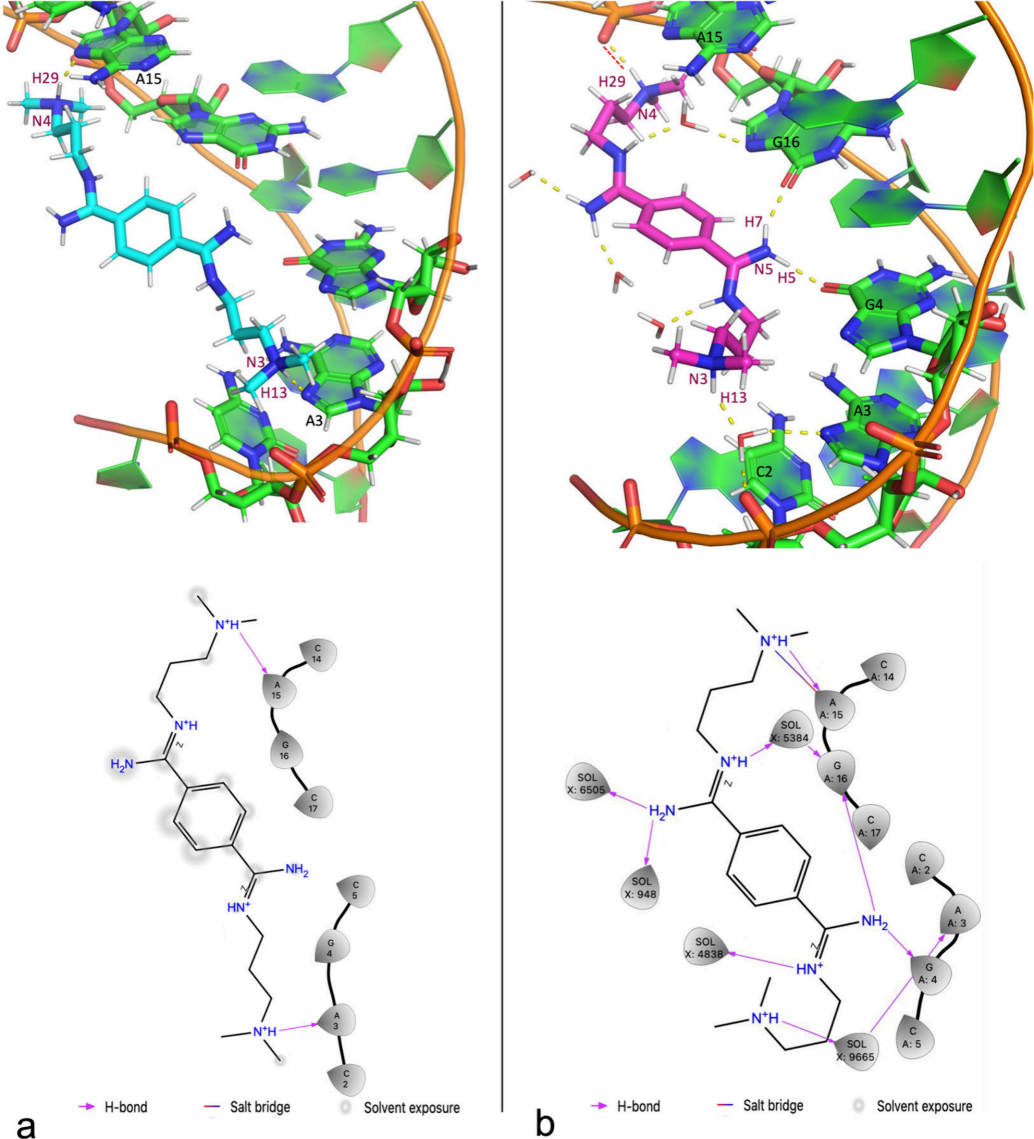
(a) Intermolecular interactions between the ligand and CAG RNA in its first (out of five) poses deposited in the PDB.^18^ Upper: 3D structure, H-bonds are drawn as yellow dashed lines. Lower: DB213 CAG RNA interaction diagram: The hydrogen atoms H13 and H29 of the ligand form hydrogen bonds with N7@A3 and N7@A15. (b) Interactions between ligand DB213 and CAG RNA in QM/MM MD. Upper: 3D structure; H-bonds are drawn as yellow dashed lines and salt bridges as red dashed lines. Lower: DB213-CAG RNA interaction diagram.

We conclude that the QM/MM simulation reproduces the experimental binding pose fairly well while also improving the binding interactions between ligand and RNA. This improvement results from slight changes in the binding pose that increase both the amount and strength of intermolecular interactions.

Notably, our QM/MM structure agrees well with the NOE data used by Peng et al. for the NMR structure refinement^18^ (Table S2). The predictions of chemical shift changes of the ligand on passing from the aqueous solution to the RNA bound state are consistent with the experiments performed here (within the statistical errors, see section 3.2 in Supporting Information, Figure S2, Table S3).

The changes in atomic charges (ΔQ) on passing from vacuum to the bound state, averaged over 250 QM/MM MD snapshots, are obtained from the electronic densities (Section 3.1 in the Supporting Information). The hydrogen atoms exhibit the largest ΔQ values with the largest fluctuations (Figure 5a). This can be seen also by a plot of the redistribution of electronic density on passing from vacuum to the RNA-bound state (Figure 5b).

**Figure 5:**
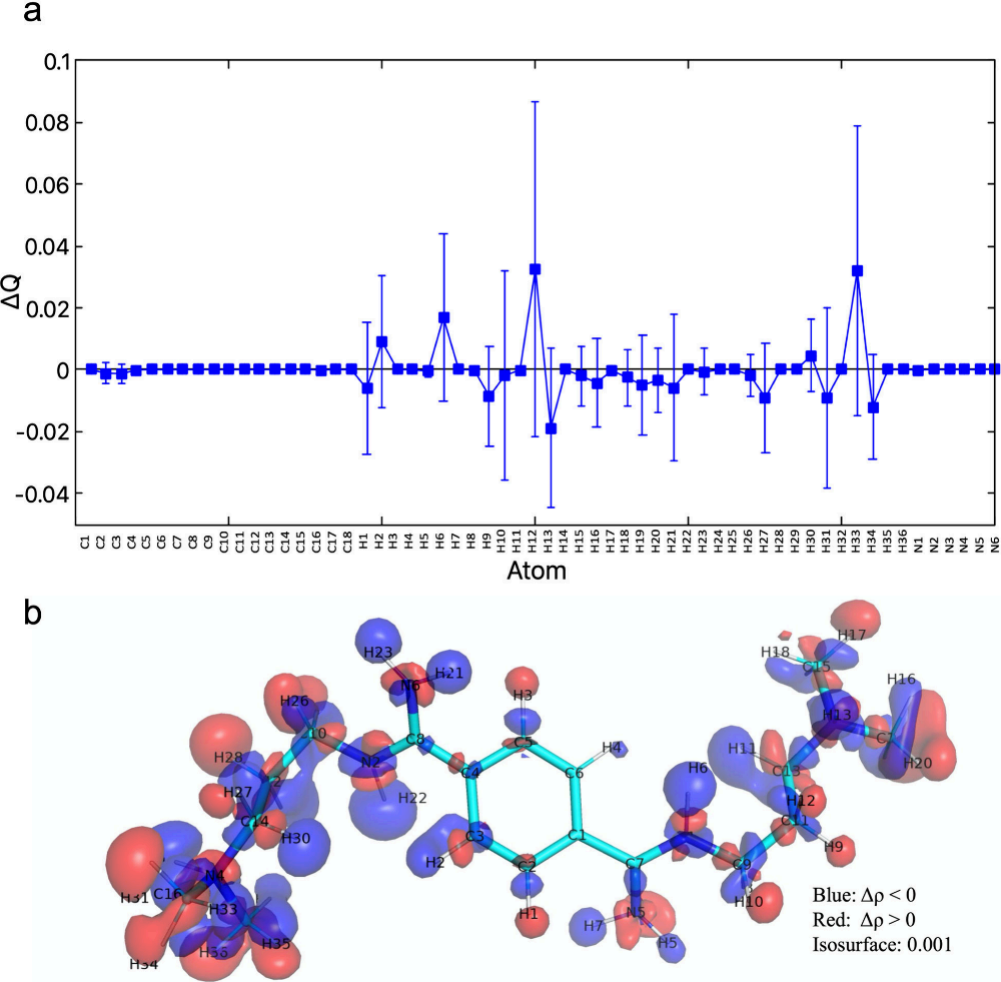
(a) Change in atomic charge for each atom (with average value and deviation) during the last 60 ps of QM/MM dynamics. (b) Change of electronic density of the ligand on passing from in vacuum to RNA-bound state. The structure is taken from a snapshot of the simulation. Δρ is the electronic density difference (RNA – vacuum). Here we present the density at the isosurface 0.001, in which blue regions indicate Δρ < 0, while red regions indicate Δρ > 0.

The overall polarization of the ligand is small yet significant. Using the atomic charge transfers values ΔQ reported in Figure 5a, we computed the total positive (negative) charge transfer ΔQ(+) (ΔQ(−)), which is defined as the sum of all the strictly positive (negative) ΔQ values. We found ΔQ(±) = ± 0.1 ± 0.08e, corresponding to a net charge transfer of ΔQ_CT_ = ΔQ(+) + ΔQ(−) = 0, as expected. The overall change in the charge distribution due to polarization effects, ΔQ_pol_, can be quantified by the sum of the absolute values of total positive and negative charge transfers, ΔQ_pol_ = |ΔQ(+)| + |ΔQ(−)| = 0.2e.

For this specific system, the value of ΔQ_pol_ is found to be of the same order of magnitude as observed in ligand/protein complexes recently studied in our group.^12,17^ Similar conclusion can be drawn for the ligand in water (Figure S3); however, the overall polarization of the ligand on passing from vacuum to water solution is smaller (ΔQ(±) = ± 0.06 ± 0.08e).

Finally, we investigate how a classical MD performs in the same time range. We find that (i) the fluctuations of the ligand during the classical MD dynamics are larger than those during the QM/MM MD (See section 5 in the Supporting Information). (ii) The ligand forms the same three H-bonds with CAG RNA as in the QM/MM but two of them are far weaker. (iii) The classical MD does reproduce the chemical shifts as the QM/MM but it shows far more NOE outliers than the latter. We conclude that the QM/MM simulation refines the NMR structure more accurately than the classical MD one.

In closing this perspective, we stress that the accurate determination of the structural dynamics of ligands is essential to advance the rational design of small molecules that interfere with RNAs. NMR is the only experimental technique that provides an ensemble of structures of RNA/drug adducts based on NOE distance constraints. Unfortunately, these are not directly measured quantities but rather arbitrarily derived from NMR. As a result, these structures may suffer from several drawbacks, some of which are discussed in the Introduction. Force field MD can be used to investigate the dynamics of the ligands and possibly improve the structural predictions, but the lack of electronic polarizability in standard force field-based MD might pose the issue of accuracy in the predictions, especially considering that RNA is a polyanion and the targets are (often more than monovalent) cations. First-principle QM/MM MD automatically incorporates electronic polarization effects and its modern versions on parallel machines can routinely reach sub-nanosecond time scales. Here we have used such a method to study the binding of the ligand DB213 to CAG RNA. The calculations show that polarization effects are indeed significant, with an overall polarization on the ligand of about one-fifth of an electron. Most importantly, they are consistent with the NMR-derived distance constraints, as well as the NMR chemical shifts measured here. Our prediction allows us to suggest that the ligand experiences some mobility. We expect the observed non-negligible change in interaction pattern not to be an artifact of the QM/MM simulation, as it enhances ligand binding to CAG RNA. This approach could be readily applied to other ligand/RNA NMR structures.

Methodologies such as force matching^23,24^ can be straightforwardly used here to extend the time scale of the simulations to the multimicrosecond range. These approaches develop an apt force field that reproduces the QM/MM trajectories. We further expect scalable machine learning-assisted free energy perturbation approaches, such as the one recently developed by our team,^25,26^ to allow prediction of binding affinities at the QM/MM level, possibly greatly improving rational design of drug targeting RNAs.

## Data Availability

Data, including input and parameter files for MD and QM/MM simulations, along with NMR chemical shifts can be found at Zenodo repository: 10.5281/zenodo.14229893

## Supporting Information Available

CAG RNA in Huntington’s disease, Computational and Experimental Methods, and Supporting figures/tables (PDF)
