# Supplementary material for: Refining Ligand Poses in RNA/Ligand Complexes of Pharmaceutical Relevance: A Perspective by QM/MM Simulations and NMR Measurements
Source: J Phys Chem Lett. 2025 Feb 10;16(7):1702–8. doi: 10.1021/acs.jpclett.4c03456 (PMC11849026; doi:10.1021/acs.jpclett.4c03456)
Supplement: Supplementary file 1 — jz4c03456_si_001.pdf [file jz4c03456_si_001.pdf]

Supporting Information:

Refining ligand poses in RNA/ligand complexes of pharmaceutical relevance: a perspective by QM/MM simulations and NMR measurements

*Gia Linh Hoang[1]**, Manuel Röck[2]**, Aldo Tancredi[2], Thomas Magauer[2], Davide Mandelli[3], Jörg B. Schulz[1,4], Sybille Krauss[5], Giulia Rossetti[3,4,6], Martin Tollinger[2]*, Paolo Carloni[1,3]**

*** contributed equally to this work.*

[1] JARA-Brain Institute Molecular Neuroscience and Neuroimaging (INM-11), Forschungszentrum Jülich, 52425 Jülich, and RWTH Aachen University, 52056 Aachen, Germany

[2] Institute of Organic Chemistry and Center for Molecular Biosciences Innsbruck (CMBI), University of Innsbruck, Innrain 80/82, 6020 Innsbruck, Austria

[3] Institute for Neuroscience and Medicine (INM-9), Forschungszentrum Jülich, 52425 Jülich, Germany

[4] Department of Neurology, Medical Faculty, RWTH Aachen University, 52074 Aachen, Germany

[5] Institute of Biology, University of Siegen, 57076 Siegen, Germany

[6] Jülich Supercomputing Center (JSC), Forschungszentrum Jülich, 52425 Jülich, Germany

Corresponding Author

* Paolo Carloni: p.carloni@fz-juelich.de

* Martin Tollinger: Martin.Tollinger@uibk.ac.at

1. RNA CODING FOR THE HUNTING PROTEIN IN HUNTINGTON'S DISEASE

Huntington's disease (HD) is an autosomal dominant disorder that leads to motor, cognitive and behavioral symptoms[1]. It belongs to the CAG triplet repeat expansion diseases, a very important group of genetically caused neurodegenerative diseases[2, 3]. It was the first identification of a CAG triplet repeat expansion disorder[4], resulting in prolonged glutamine chains (PolyQ disorders). HD drives neuronal dysfunction and death by many molecular mechanisms, involving the human mutant HTT gene. The healthy gene contains less than 35 CAG repeats, whereas more than 40 repeats in the mutant gene invariably cause the disease.

Mutant HTT encodes a HTT protein with an abnormally expanded polyglutamine tract. This forms aggregates in patients' brains[2, 5-7], a hallmark of the disease[8]. Mutant HTT gene products cause diverse cellular pathomechanisms both inside and outside the nucleus. Thus, a compound that would block mutant HTT RNA in the cytoplasm will still inhibit these pathomechanisms (including aberrant translation of HTT via the MID1 complex). In the mutant HTT RNA, the CAG repeat (mutCAG RNA here after) folds into a hairpin structure absent in healthy HTT RNA[9-12]. Such hairpin structures abnormally capture RNA-binding proteins (such as the protein MID1 or G-rich RNAs[13] **(Figure S4)**) resulting in RNA-mediated cellular dysfunction. RNA-mediated abnormal processes include translation initiation of the mutCAG RNA (by trapping parts of the translation machinery) as well as aberrant splicing (by trapping splice factors)[14-17] **(Figure S5)**. Importantly, RNA toxicity is only triggered by expanded CAG repeats but not by other trinucleotide repeats encoding also for glutamine (i.e., CAA)[18-20]. Ligands interfering with mutCAG RNA function and interactions with its cellular partners may provide important insight into the impact of mutCAG RNA on the development of HD. They may also constitute promising therapeutic agents.

2. RECENT POLARIZABLE FORCE FIELD STUDIES ON RNA

For the last two years, two papers have been published on polarizable force field - based MD of RNAs oligonucleotides with a number of nucleobases 10 or longer. In ref.[21], AMOEBA force field[22, 23] - based molecular dynamics (MD) simulations of the different protein/RNA complexes were not always able to reproduce the experimental structural determinants.

In ref. [24], milestoning simulations predicted the energy landscape of an RNA 10-mer in solution. The authors found that A-RNA is lower in energy than B-RNA. Their calculations also showed that this oligomer is highly flexible.

3. METHODS

3.1. Simulations

Force field-based MD simulations. Our calculations are based on the first of the five NMR structures of the CAG RNA-DB213 complex, (PDBid 7D12)[25], using the first conformer. We considered two systems: (i) The CAG RNA-DB213 complex, which was embedded in a cubic box of edge 8.7 nm, filled with 21,463 water molecules. 15 Na^+ ions were added to neutralize the RNA/ligand complex and 14 Na^+ and 14 Cl^- ions were added to the system to mimic the experimental conditions of the NMR experiments (pH 7.0, 35mM NaCl) **(Figure S6)**. The shortest distance of the complex from the box's edges was set to 2.2 nm to minimize the interactions between periodic replicas. (ii) the DB213 ligand in water solution. The ligand was embedded in a cubic box of edge 8.4 nm containing 18,988 water molecules. 4 Cl^- ions were added to neutralize the system and 12 Na^+ and 12 Cl^- ions were added to achieve the same conditions of the experiments as (i).

The force fields for RNA, ions, and water were the AMBER-parmbsc0[26], the Joung-Cheatham[27] and the TIP3P[28], respectively. The partial atomic charges of the ligands were set equal to the corresponding RESP charges[29] calculated at the B3LYP/6-31G(d,p) level of theory using the Gaussian 09 package[30] and the bonded terms of the ligand were treated with General AMBER Force Field (GAFF)[31].

We used an integration time step of 2 fs. All bonds to hydrogen atoms were constrained using the LINCS algorithm[32]. The non-bonded interactions (Coulomb and van der Waals) were computed using the Particle-Mesh Ewald method[33, 34], using a grid spacing value of 1.2 Å and a short-range cutoff of 12 Å. For the system (i), the Nose-Hoover thermostat[35] and isotropic Parrinello–Rahman barostat[36] were used for keeping temperature and pressure constant. For the Nose-Hoover thermostat, we used a time constant of 0.5 ps and a reference temperature of 298K. For the Parrinello–Rahman barostat, we used an isotropic pressure coupling with a time constant of 5 ps, using the isothermal compressibility of water (4.5e^{-5} bar^{-1}) and a target pressure of 1 bar. To maintain constant temperature and pressure of the system (ii) with the same temperature and pressure as (i), we used velocity rescaling with a stochastic term thermostat[37] with a time constant of 0.1 ps, and an isotropic Berendsen barostat[38] with a time constant of 2 ps and the isothermal compressibility of water as 4.5 10^{-5} bar^{-1}.

The system (i) underwent energy minimization using both the steepest descent[39] and the conjugate gradient[40] algorithms, until the maximum force on each atom was 24 kcalmol^{-1}nm^{-1} or lower. Then, 4 ns-long MD simulation was performed applying harmonic restraints (spring constant of 240 kcal mol^{-1}nm^{-2}) to the atoms of the complex atoms to keep it close to the reference configuration obtained at the end of the minimization. We used this simulation to gradually heat the entire system up to the target temperature of 298 K through 9 intermediate temperature points (0,40,80,120,160,200,240,280,

and 298 K). After the heating phase, a 1 ns-long NVT equilibration was performed at T=298 K, followed by a 1 ns-long NPT simulation at the same temperature and 1 bar pressure. These equilibration phases were performed with the same harmonic restraints applied to the complex. The system (ii) underwent the same energy minimization process as (i) and then was heated and equilibrated at the same temperature and pressure as (i) in a 1 ns-long equilibration.

All the force field-based MD simulations were performed using the GROMACS/2021.7 (https://doi.org/10.5281/zenodo.7586728) suite of codes.

QM/MM MD simulations. The last snapshots obtained from the classical MD simulations of the ligand in complex with CAG RNA (i) and in water (ii) were used as the starting structure to set up our QM/MM MD simulations. The total system was partitioned into a QM region, including only the ligand (60 atoms), and a MM region, including CAG RNA, water and ions. The QM region was treated at the BLYP level of theory[41, 42] as implemented in the planewaves density functional theory (DFT) code CPMD[43], using a cut-off of 70 Rydberg for the expansion of the Kohn-Sham orbitals. Only valence electrons were considered, using Troullier-Martins norm-conserving pseudopotentials[44], and the Martyna-Tuckerman's method[45] was used to achieve isolated system conditions. The interactions between the QM and MM subsystems were computed using the MiMiC implementation[46] of the electrostatic embedding scheme by Laio et al.[47]. In our simulations, we used a cut-off distance of 70 a.u (3.7 nm) for the short-range interactions and a multipole order of 7 for the long-range coupling. The MM region was treated in GROMACS. The QM region underwent Born-Oppenheimer MD[48]. An integration timestep of ~0.24 fs was used for the entire system.

The geometry of both systems (i) and (ii) were first optimized by simulated annealing using a damping factor of 0.95 until the temperature of the system reached below 5 K. This was followed by a gentle

heating protocol where the temperature was gradually raised to 298 K in 2.4 ps for (i) and in 7.2 ps for (ii) in the NVT ensemble using the Berendsen-type thermostat[38] with the time constant τ =3000 a.u (~73 fs). Finally, a 14.4 ps-long equilibration phase at 298 K was performed where the temperature of the system was controlled by applying two independent Nose-Hoover chains[49-51] thermostats to the QM and MM regions. The same thermostat settings were used for both QM and MM region, namely, Nose-Hoover thermostat with the same desired temperature at 298 K and the thermostat frequency at 4000 cm^{-1}. The last configuration obtained from the NVT equilibration run was used as the starting point for a final 100 ps-long production run for system (i) and 72 ps-long production run for (ii) in the NVT ensemble using the same set up. The MiMiCPy package[52] was used to prepare all the GROMACS and CPMD input files required to run the QM/MM MD simulations.

The following properties were calculated:

(a) The electronic polarization[53] is described here in terms of changes of electronic densities and of atomic charges as in ref.[54], using the Voronoi partition scheme[55]. From the change of electronic density of the ligand on passing from in vacuo to in binding with RNA, we calculated the integral over the grid points within the Voronoi partition to obtain the change in atomic charge for each atom (ΔQ) of the ligand. A python code, cpmd-cube-tools[56], was used for this calculation.

(b) NMR chemical shift calculations were performed using a QM/MM scheme based on the ORCA program package[57, 58]. The NMR chemical shielding tensors were computed using Coupled Perturbed Self Consistent Field (CP-SCF) calculations. The Gauge-Independent Atomic Orbitals (GIAOs) were employed to address the problem of result's dependence on the choice of gauge origin[59]. The resolution of the identity approach (RI) was used to approximate the Coulomb term, while exchange contributions are approximated according to the RIJONX scheme[57, 59]. Our calculations were

performed within the DFT with a meta-GGA functional (M06-L), along with pcSseg-2 basis set, which is an atom-centered Gaussian-type orbital (GTO) basis specialized for the chemical shielding calculation and an auxiliary basis set def2/JK. We calculated the changes in ^{1}H (ΔH) and ^{13}C (ΔC) chemical shifts of the ligand on passing from water to the RNA bound state.

The properties (a) and (b) were calculated over 250 snapshots (last 60 ps).

3.2. Experiments

CAG RNA was prepared by solid phase synthesis, desalted, and purified using preparative anion exchange chromatography as described previously[60]. The purity of the RNA was verified by anion exchange chromatography, and ^{1}H NMR spectroscopy and the concentrations of all samples were determined by measuring the UV absorption at 260 nm. Ligand DB213 was synthesized following a reported procedure with slight modifications (see the section below for details)[25, 61]. Although the initial purity was satisfactory, an additional reverse-phase chromatography step was performed to obtain a higher-purity sample, as verified by mass spectrometry and NMR spectroscopy (**Figure S7**).

All NMR experiments were performed at a temperature of 25 °C using a 700 MHz NMR spectrometer equipped with a Prodigy TCI cryogenic probe (Bruker Biospin, Germany). NMR samples for recording the chemical shifts of DB213 contained 200 μM DB213 dissolved in 10% D_2O/90% H_2O, 35 mM NaCl. Chemical shifts of DB213 bound to RNA were obtained using a sample that additionally contained a small excess (220 μM) of CAG RNA. All samples contained 2,2-dimethyl-2-silapentane-5-sulfonate (DSS, 4 μM) as internal chemical shift reference for ^{1}H and ^{13}C. ^{1}H chemical shifts were recorded in 1-dimensional experiments using excitation sculpting for water suppression[62], and ^{13}C chemical shifts were obtained from 2-dimensional sensitivity-enhanced $^{1}H^{13}C$ HSQC experiments. All NMR spectra were processed and analyzed using Bruker Topspin 4.4.1.

Synthesis of DB213. Acetyl chloride (4.0 mL) was added dropwise to dry ethanol (5.0 mL) at 0 °C (exothermic). After 5 minutes, 1,4-dicyanobenzene (0.500 g, 3.90 mmol, 1 equiv) was added to the reaction mixture at 0 °C, and the reaction mixture was allowed to warm to 23 °C. After 12 hours, the resulting white suspension was filtered under vacuum and washed with dry ethanol (1×15 mL) and dry diethyl ether (2×15 mL). The white solid was resuspended in dry ethanol (30.0 mL), and Et_3N (1.63 mL, 11.7 mmol, 3.00 equiv) was added to give a clear yellow solution followed by addition of N,N-dimethyl-1,3-diaminopropane (1.22 mL, 9.76 mmol, 2.50 equiv), and the resulting solution was stirred at 23 °C. After 24 hours, the volatiles were removed under vacuum, and the yellow oil was resuspended in dry diethyl ether (2×15 mL) and filtered twice. The resulting white solid was dried under high vacuum, and a freshly prepared mixture of ethanol (5.0 mL) and acetyl chloride (4.0 mL) was slowly added at 0 °C (exothermic). The reaction mixture was heated at 60 °C for 3 hours, cooled to room temperature, and centrifuged (4,000 rpm, 5 min) to precipitate the target compound. The precipitate was subsequently washed with dry ethanol (1×10 mL) and dry diethyl ether (2×10 mL) to give DB213 (390 mg, 0.838 mmol, 21%) as a white solid.

An analytically pure sample (~95% purity) of DB213 was obtained via reverse-phase semi-preparative HPLC on an ÄKTAprime Plus system with a puriFlash® RP-AQ column. A solution of ammonium hydroxide in water (30% w/w) was added, and the corresponding free amine was injected onto the column at a flow rate of 20 mL/min. Elution was performed with water over 30 minutes to remove impurities. Subsequently, the corresponding HCl salt was eluted with 0.1% HCl, collected, and dried under reduced pressure. The obtained analytical data was in full agreement with those reported in the literature[25, 61].

TLC (silicagel 60 RP-18 $F254_s$): (10% ACN in 0.1% HCl): R_f=0.12 ($KMnO_4$, UV); ^{1}H NMR (700 MHz, DMSO-d_6): δ 7.97 (s, 4H), 3.54 (t, J=7.0 Hz, 4H), 3.19–3.15 (m, 4H), 2.76 (s, 12H), 2.10–2.04

(m, 4H). ^{13}C NMR (176 MHz, DMSO-d_6): δ 162.5, 133.1, 129.0, 54.3, 42.5, 40.3, 22.7. MS (ESI) calc. for $C_{18}H_{33}N_6$ $[M+H]^+$: 333.2761; found: 333.2759.

4. INTERMOLECULAR INTERACTIONS BETWEEN THE LIGAND AND CAG RNA

The ligand exhibits two positively charged tails (each bearing a partial charge of +0.8 according to our RESP charges calculations, **Figure S8**). These two charged tails interact (either directly or by a water bridge) with the negatively charged phosphates of A15 and C2 (Figure 4b in Main text). Such electrostatic stabilization is absent in all of the NMR complexes: indeed, the ligand tails interact with N7@A15 and N7@A3 (**Figure 4a** in Main text).

As for the ligand/RNA H-bond interactions, we notice that (i) all H-bond distances (d in **Figure S9a**) for QM/MM and NMR models are in the 1.7-1.9 Å range **(Table S4)**; (ii) all D-H… A (D, A =N or O atoms) angles (α in **Figure S9a**) are close to 180° **(Table S5)**. (iii) Some H-bond interactions in the NMR models involving a nitrogen in an aromatic ring exhibit a high deviation of the hydrogen bond from the aromatic ring plane (angle θ in **Figure S9b**). In a "strong" H-bond with N-atoms of the nucleobases as acceptors, θ values are less than a couple of dozen degrees (25°-30° according to ref.[63] and 25° (with few outliers with θ=30° or 40°) in ref.[64]). Indeed, H-bonds are formed in the direction of the acceptor's electron pair[63]. In the N7@A15 …H29-N4 and N7@A3 … N3-H13 interactions, θ ranges from 44° to 62° **(Table S6)**, and therefore these interactions may be considered as weak H-bonds.

Table S4-S6 and **Figure S10** show that three strong H-bonds present in the QM/MM dynamics replace (i) two weak H-bonds in the NMR 1 structure, (ii) 1 strong H-bond and two weak H-bonds in

the NMR 2, NMR 4, NMR 5 structures, (iii) 2 strong H-bonds and two weak H-bonds in the NMR 3 structure.

We conclude that, at the qualitative level in the QM/MM simulations, the CAG RNA/ligand electrostatic interactions may be stronger than that in the NMR 1 structure, that is the model we started the QM/MM simulations with. In addition, in the QM/MM simulations, two weak H-bonds are replaced by three strong H-bonds. Thus, overall, the QM/MM pose does form stronger interactions with CAG RNA than that of the NMR1 pose.

Is this the case in the other NMR structures? For NMR2,4,5 structures, the two weak H-bonds along with one strong H-bond are replaced by three strong H-bonds in the QM/MM dynamics. In addition, the CAG RNA/ligand electrostatic interactions remain weaker as in the first NMR model. Thus, also in these cases we expect stronger interactions for the QM/MM pose.

In the case of the NMR3 structure, the issue is more difficult: Indeed, two weak and two strong H-bonds are replaced by three strong H-bonds in the QM/MM MD, whereas the RNA/ligand electrostatic interactions remain weaker for the NMR structure.

5. CLASSICAL MD

100 ps classical MD simulation are here performed based on the same force field used for the MM part of the QM/MM simulation.

The RMSD values of the backbone atoms relative to the initial NMR structure oscillates around 0.15 nm **(Figure S11a)**, as in the QM/MM simulations. The regions that exhibit the largest fluctuations are, as expected, those at the termini and the loop. In the region near the ligand's binding pose, C5,

A15, G16, C17 are relatively rigid (as in the QM/MM simulations). However, A3 and G4 have larger fluctuations **(Figure S11b)** than those in the QM/MM dynamics (**Figure 2b** in the main text).

The ligand RMSD, calculated as in the QM/MM simulation is larger than in QM/MM dynamics **(Figure S12** and **Figure 3** in the main text, respectively). We analyzed the last 60 ps, as in the QM/MM simulation. The H-bonds H5…O6@G4, H7…O6@G16 and H29…OP2@A15 are formed as in the QM/MM simulations, but the first two are much weaker **(Table S7** and **Table S1**, respectively**)**. Hydrophobic interactions are formed between the ligand and C2, A3, G4, C14, A15, G16 of CAG RNA, as in the case of QM/MM simulation and the NMR structure.

The structures from classical MD simulation shows more NOE outliers (6 outliers) than structures from QM/MM (2 outliers) **(Table S8** and **Table S2**, respectively**).**

The predictions of chemical shift changes of the ligand on passing from the aqueous solution to CAG RNA bound state in classical MD are consistent with the experiments within the statistical errors, as in the case of QM/MM simulation **(Table S9** and **Table S3**, respectively).

6. FIGURES AND TABLES

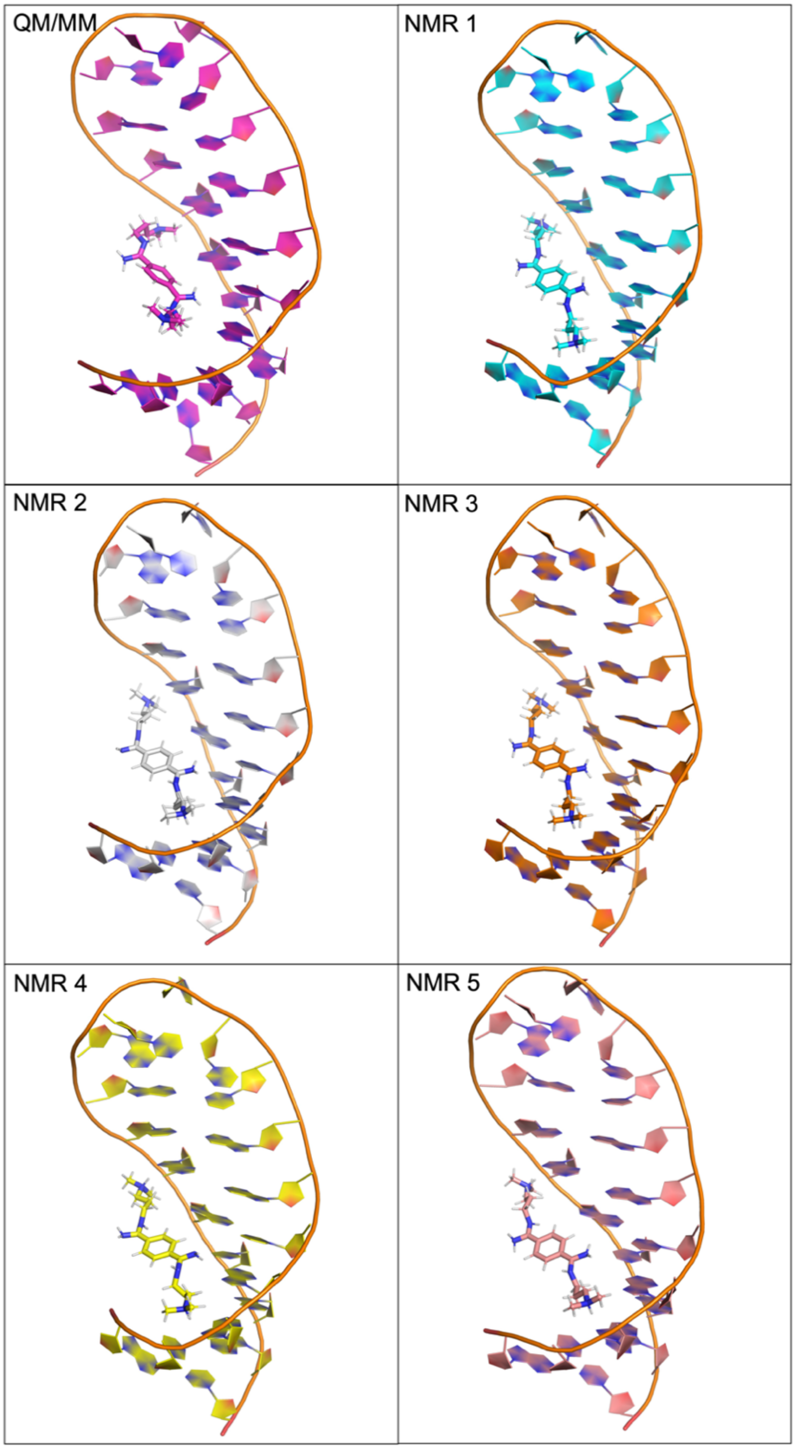

Figure S1. QM/MM structure of the CAG RNA/ligand complex after 100ps along with and the 5 structures from NMR[25].

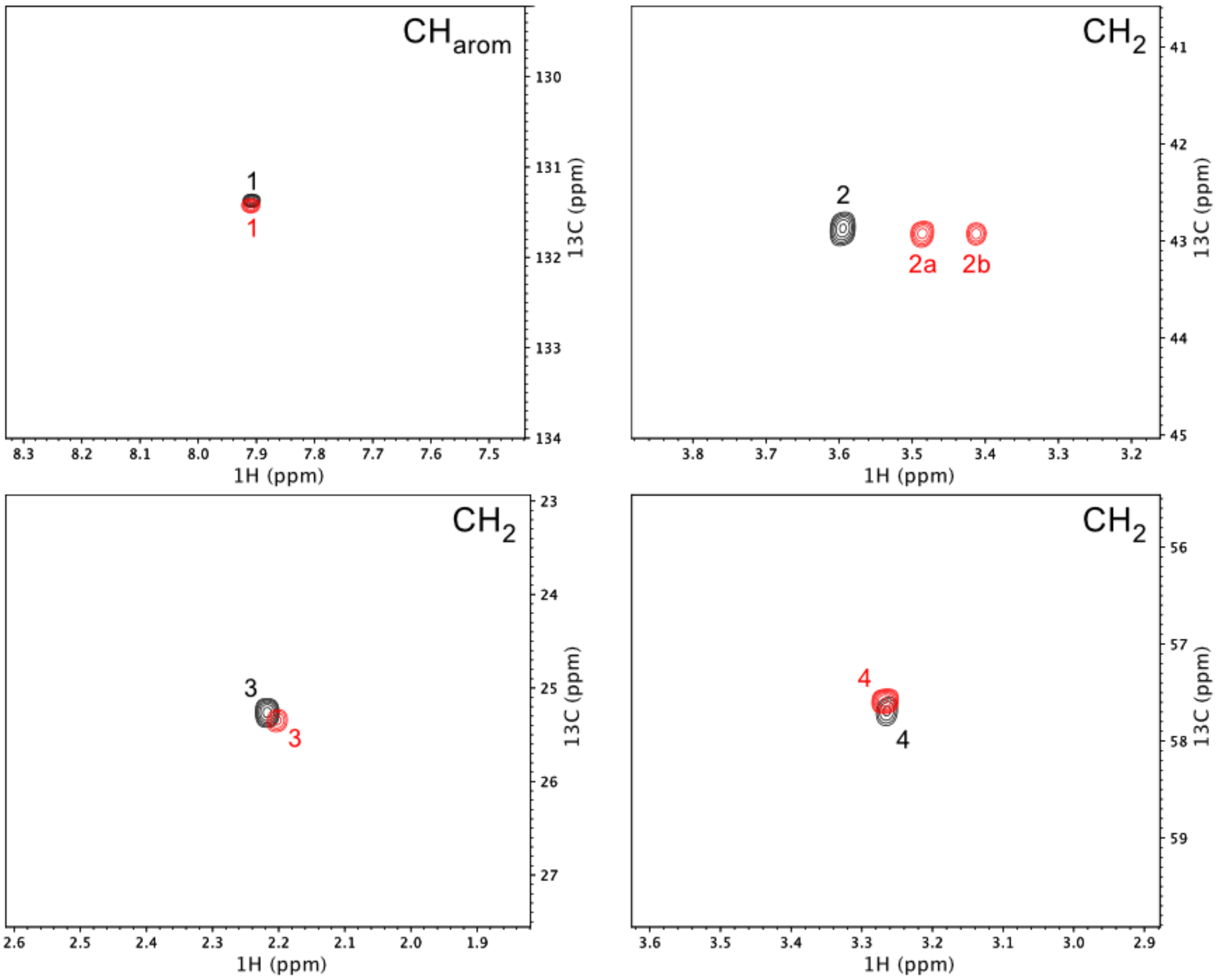

Figure S2. ^{1}H and ^{13}C chemical shift changes observed for ligand DB213 upon CAG RNA binding. Sections of 2-dimensional sensitivity-enhanced ^{1}H^{13}C HSQC spectra recorded for 200 μM DB213 in 10% D_2O/90% H_2O, 35 mM NaCl, in the absence (black) and in the presence (red) of 220 μM CAG RNA. DB213 resonances are labeled according to Chart 1 (main text).

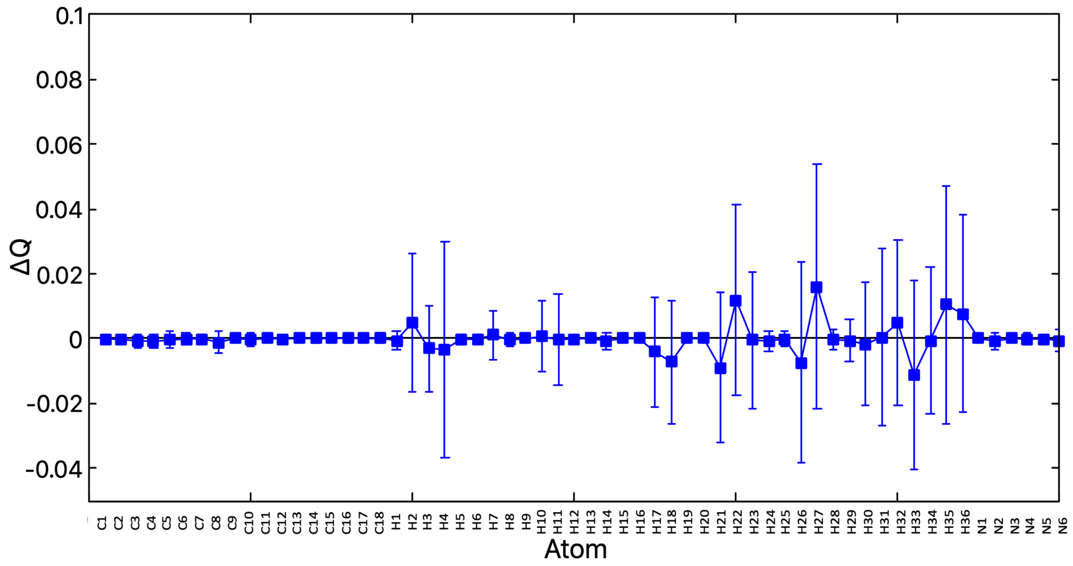

Figure S3. Change in atomic charge for each atom of the ligand (with average value and deviation) during the last 60 ps of QM/MM dynamics of ligand in water.

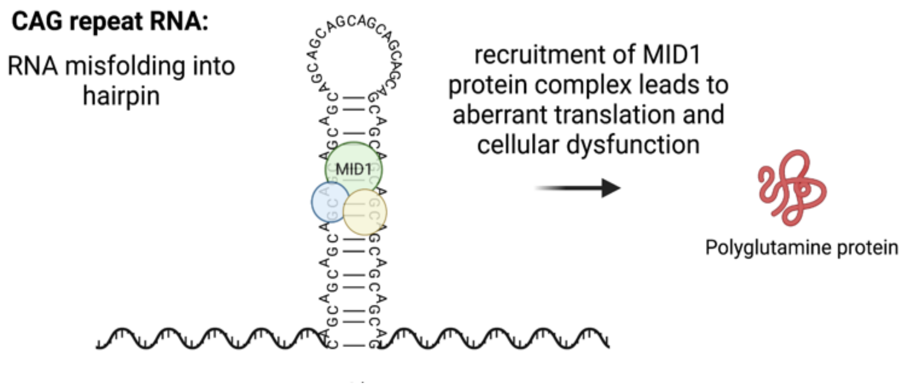

Figure S4. MutCAG RNA folds into a hairpin, which abnormally captures RNA-binding proteins (such as the protein MID1), resulting in cellular dysfunction.

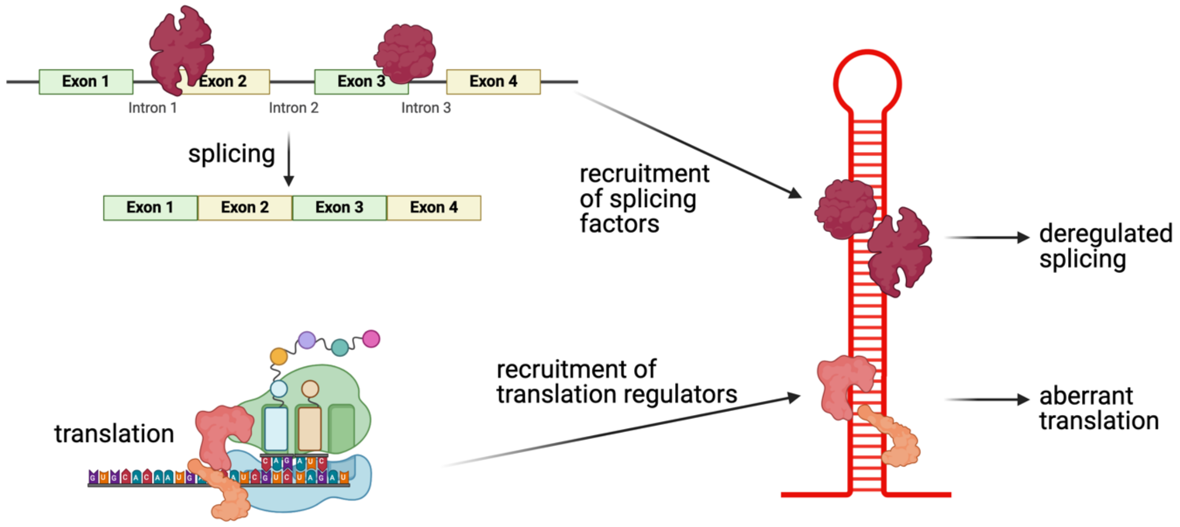

Figure S5. MutCAG RNA hairpins sequester several proteins that carry out aberrant activities in conjunction with the RNA-hairpin. These activities include aberrant translation and deregulated splicing. Figure adapted from[65], created with biorender.com.

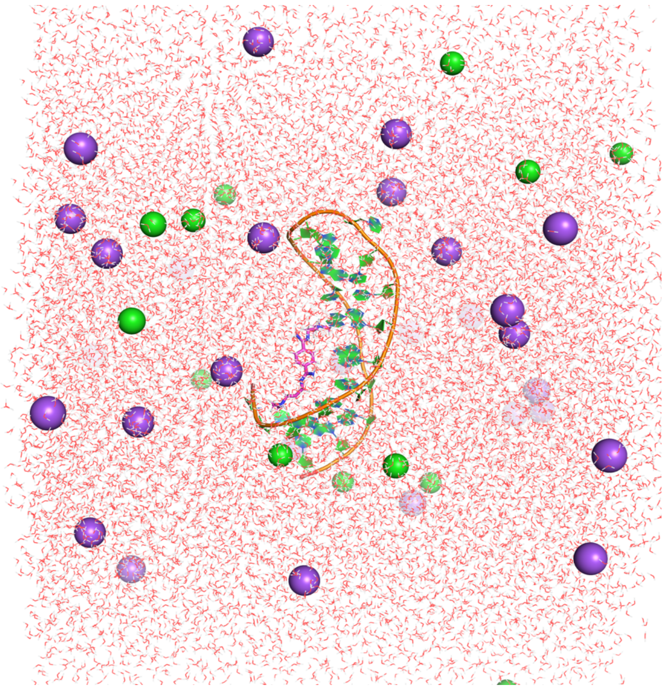

Figure S6. Simulation box for the DB213 -CAG RNA complex in explicit solvent. CAG RNA is shown in cartoon presentation, the ligand as sticks, the Na+ ions as violet spheres, the Cl- ions as green spheres.

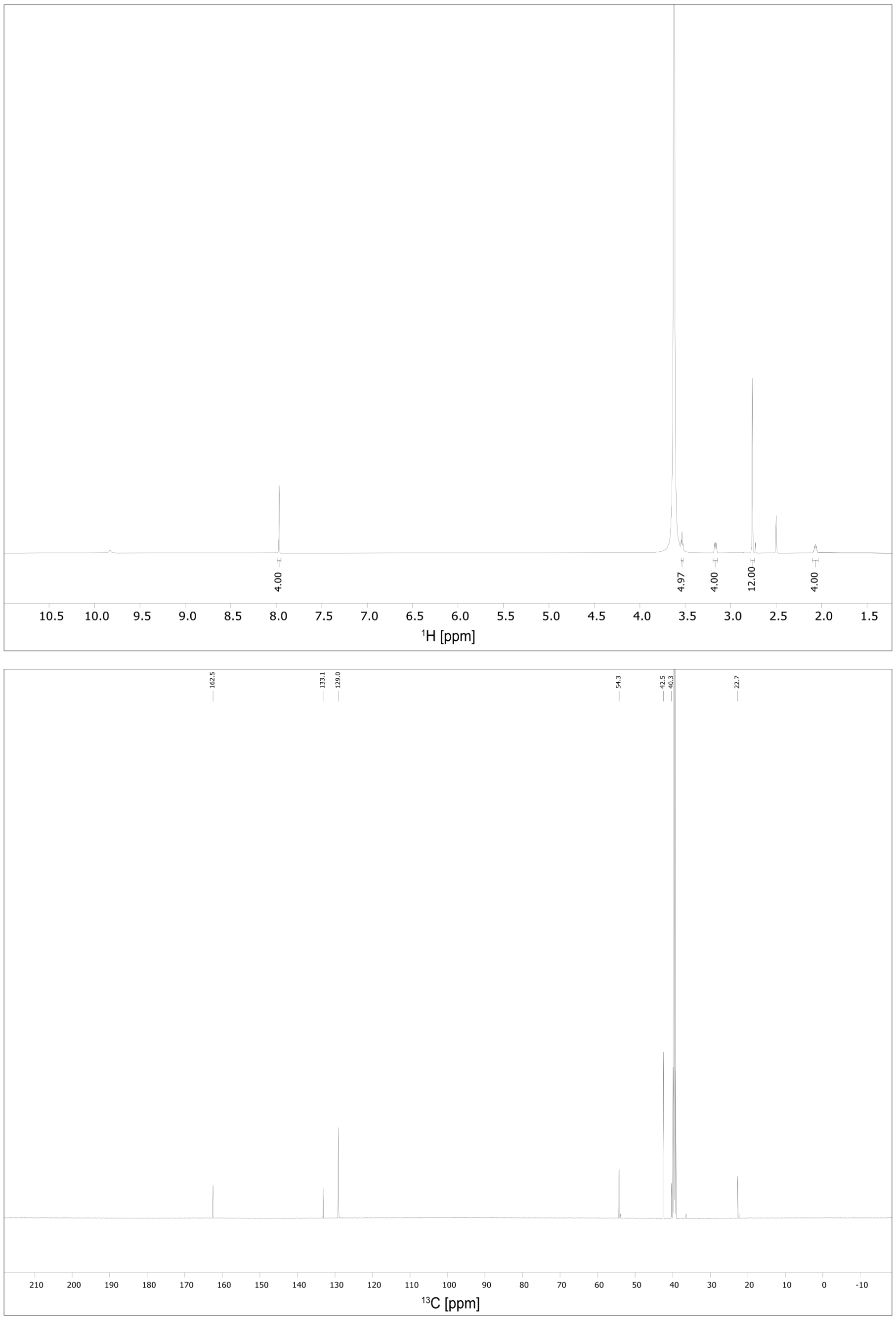

Figure S7. ^{1}H NMR (top) and ^{13}C (bottom) NMR spectra of DB213 dissolved in DMSO-d_6, recorded at a temperature of 25 °C, 700 MHz.

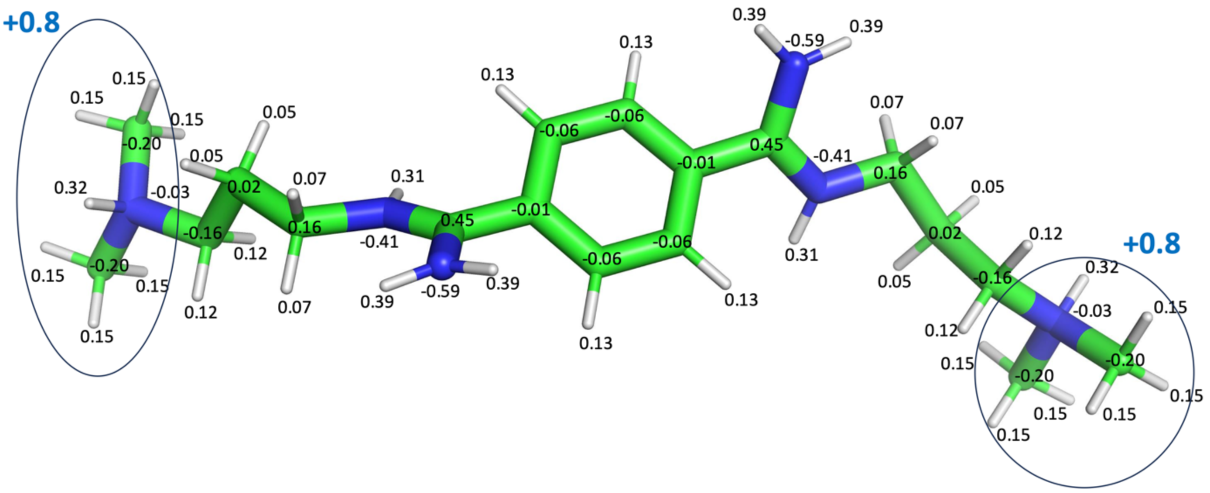

Figure S8. RESP charges of the DB213 ligand.

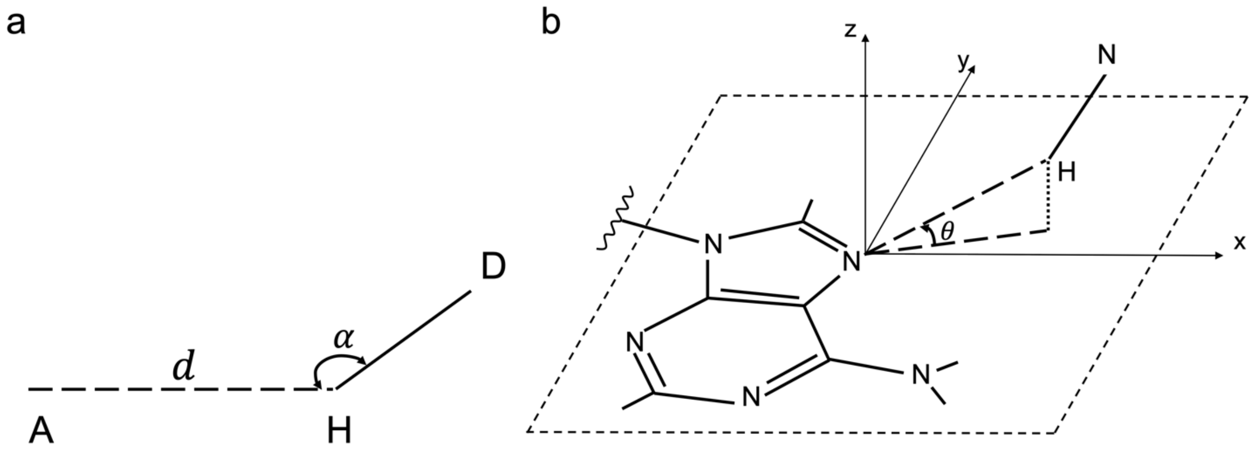

Figure S9. (a) Selected structural parameters (d, α) of a H-bond, in which D represents donor and A represents acceptor (b) Selected structural parameter (θ) of adenine's base N atom as H-bond acceptor, interacting with H-N moiety. The figure is inspired by Fig.1 in ref.[64]

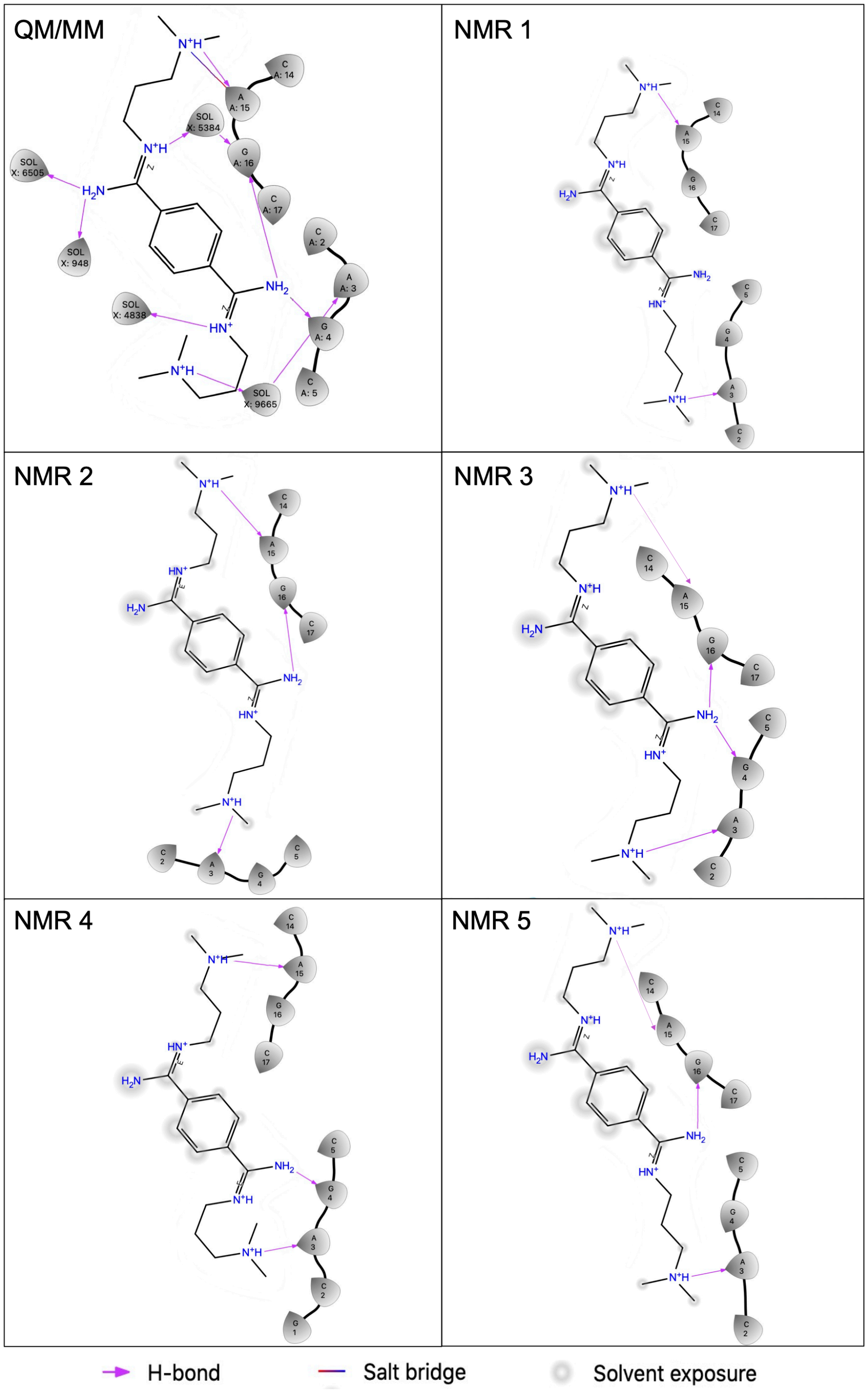

Figure S10. DB213 - CAG RNA interaction diagrams of the last QM/MM snapshot after 100ps and of the 5 structures obtained by NMR[25].

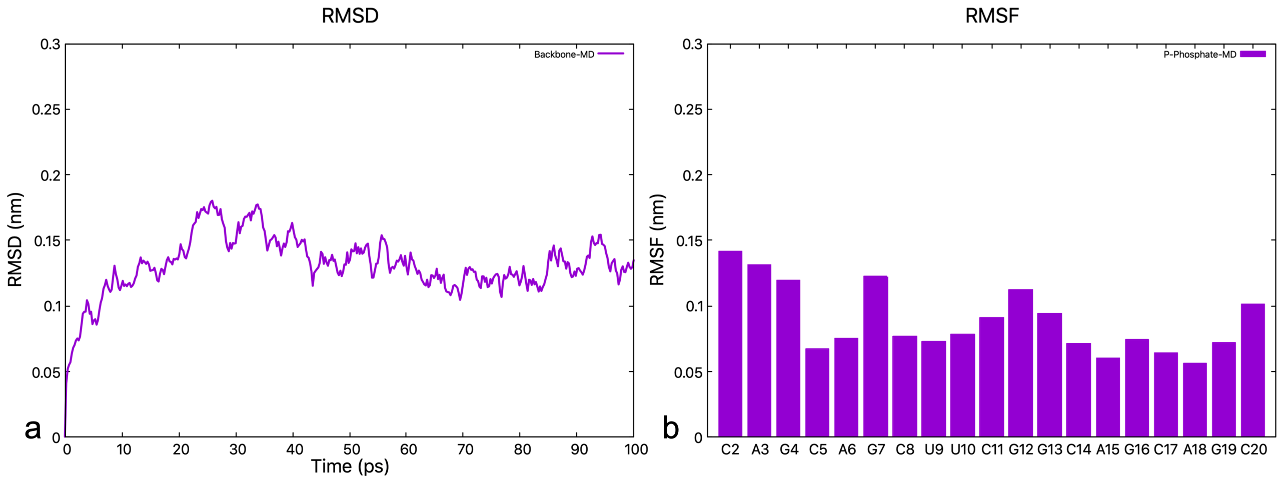

Figure S11. (a) RMSD of the CAG RNA backbone as a function of the simulated time. (b) RMSF histogram of the phosphate phosphorus atoms of each nucleobase.

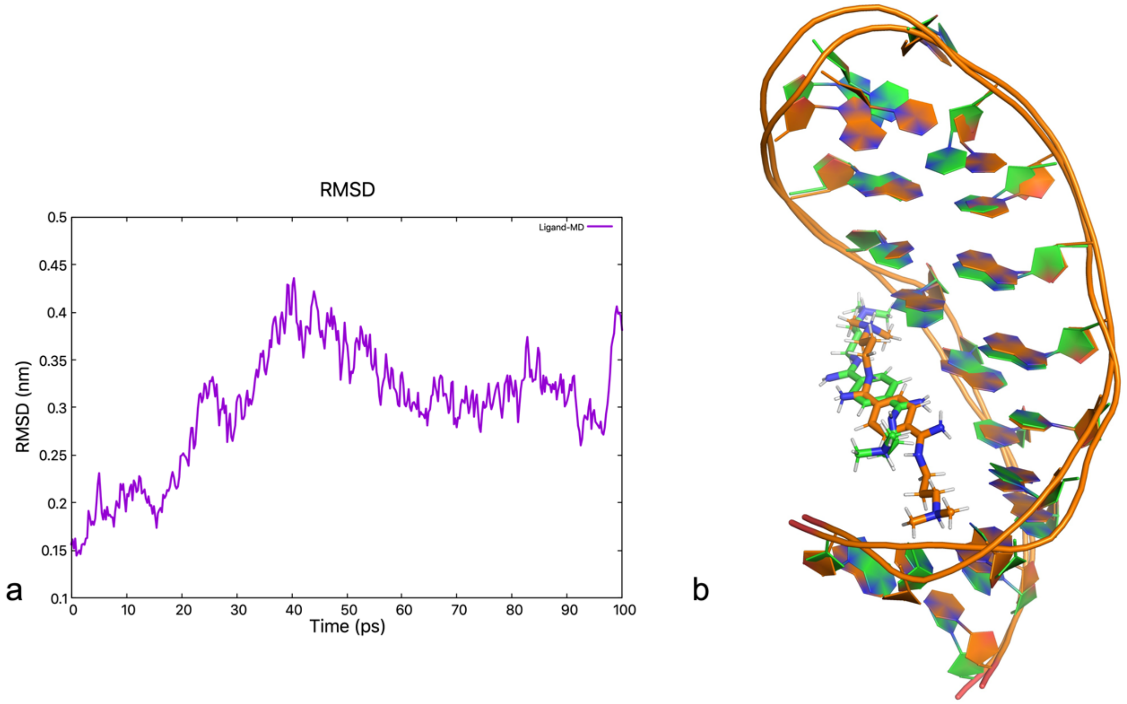

Figure S12. (a) RMSD of the ligand DB213 relative to the NMR structure as a function of simulated time during the classical MD. The CAG RNA backbone is fitted to the NMR every timestep. (b) Overlap of the last snapshot of classical MD (green) with the NMR 1 structure (orange, RMSD=0.38 nm)

Table S1. Average distances and time courses for hydrogen bonds and salt bridges during the QM/MM MD simulation.

Type of interactions	CAG RNA	DB213	Average distance (Å)	Time course (%)
H- bond	G4-O6	N5-H5	1.8 (0.09)	94.0%
H- bond	G16-O6	N5-H7	1.8 (0.09)	85.6%
H-bonded Salt bridge	A15- OP2	N4-H29	1.7 (0.09)	100.0%

Table S2. NOE structural constraints imposed by Peng et al. for the NMR structure refinement of the complex[25]. The upper and lower boundary of the distance restraints used in NMR structure refinement [Å], as derived from a long-range NOESY experiment (mixing time: 1 s) are indicated with **restr**. The distances between pairs of atoms of CAG RNA and ligand DB213 from the NMR structure (used here as a starting point of the QM/MM MD calculations) and as those obtained as averages from the QM/MM MD simulation, are noted as **NMR** and **SIM**, respectively. The standard deviations for the latter are indicated **STD**. The two symmetric parts of the ligand DB213 are colored cyan and green, and the green labels 2, 3, 4, 5 in the first column follow **Chart 1** in the main text. All intermolecular distances for a particular CH2 or CH3 group falling outside the restraint range used for NMR structure refinement are colored in red. This applies to one CH2 group in the NMR structure and two CH2 groups in the QM/MM MD simulation, which are located slightly beyond the upper limit of 9.0 Å. This value was arbitrarily chosen by Peng et al. for the majority of internuclear distances. Thus, outliers with distances exceeding this limit due to spin diffusion are to be expected.

	RNA		DB213	restr		NMR	SIM	STD	RNA		DB213	restr		NMR	SIM	STD
**CH2** 2	C2	H1'	H131	4	9	8.5	9.2	0.3	C14	H1'	H434	4	9	9.4	8.7	0.4
	C2	H1'	H13A	4	9	9.0	8.4	0.3	C14	H1'	H43A	4	9	9.5	9.8	0.3
**CH2** 3	C2	H1'	H141	4	9	6.6	6.9	0.3	C14	H1'	H444	4	9	7.3	7.5	0.3
	C2	H1'	H14A	4	9	7.4	6.9	0.4	C14	H1'	H44A	4	9	8.2	7.5	0.4
**CH2** 4	C2	H1'	H151	4	9	8.3	8.3	0.8	C14	H1'	H454	4	9	8.5	9.5	0.4
	C2	H1'	H15A	4	9	8.7	8.9	0.3	C14	H1'	H45A	4	9	9.4	9.7	0.2
**CH3** 5	C2	H1'	H171	4	9	5.8	6.5	0.9	C14	H1'	H474	4	9	7.0	9.7	0.7
	C2	H1'	H17A	4	9	5.9	6.1	0.9	C14	H1'	H47A	4	9	7.4	9.5	0.4

	C2	H1'	H17B	4	9	7.3	6.5	1.5	C14	H1'	H47B	4	9	8.6	10.2	0.4
	C2	H1'	H181	4	9	7.4	9.1	0.5	C14	H1'	H484	4	9	7.7	7.8	0.7
	C2	H1'	H18A	4	9	8.3	8.4	0.6	C14	H1'	H48A	4	9	8.2	8.9	0.6
	C2	H1'	H18B	4	9	8.6	9.1	0.9	C14	H1'	H48B	4	9	9.1	7.8	0.5
**CH2** 2	A3	H8	H131	3	7	6.7	6.3	0.3	A15	H8	H434	3	7	6.7	6.0	0.4
	A3	H8	H13A	3	7	7.1	6.2	0.3	A15	H8	H43A	3	7	6.7	6.7	0.3
**CH2** 3	A3	H8	H141	3	7	4.8	4.2	0.3	A15	H8	H444	3	7	4.4	3.8	0.3
	A3	H8	H14A	3	7	5.8	5.4	0.4	A15	H8	H44A	3	7	4.6	4.4	0.4
**CH2** 4	A3	H8	H151	3	7	4.9	6.4	0.5	A15	H8	H454	3	7	5.2	5.8	0.3
	A3	H8	H15A	3	7	5.6	6.0	0.3	A15	H8	H45A	3	7	5.4	5.6	0.3
**CH3** 5	A3	H8	H171	3	7	3.3	4.9	1.2	A15	H8	H474	3	7	3.3	5.6	0.7
	A3	H8	H17A	3	7	4.2	4.3	1.3	A15	H8	H47A	3	7	4.3	5.7	0.4
	A3	H8	H17B	3	7	4.8	4.9	1.5	A15	H8	H47B	3	7	4.8	6.1	0.4
	A3	H8	H181	3	7	3.1	5.9	0.5	A15	H8	H484	3	7	3.0	3.2	0.8
	A3	H8	H18A	3	7	3.8	5.8	0.6	A15	H8	H48A	3	7	3.7	4.2	0.5
	A3	H8	H18B	3	7	4.6	6.6	0.5	A15	H8	H48B	3	7	4.5	3.3	0.5
**CH2** 2	A3	H1'	H131	4	9	8.3	8.6	0.3	A15	H1'	H434	4	9	8.1	9.0	0.4
	A3	H1'	H13A	4	9	9.3	8.1	0.4	A15	H1'	H43A	4	9	8.9	9.7	0.3
**CH2** 3	A3	H1'	H141	4	9	7.3	6.7	0.3	A15	H1'	H444	4	9	6.1	6.7	0.3
	A3	H1'	H14A	4	9	8.7	8.2	0.5	A15	H1'	H44A	4	9	6.7	7.8	0.4
**CH2** 4	A3	H1'	H151	4	9	6.8	9.7	0.5	A15	H1'	H454	4	9	7.8	8.8	0.2
	A3	H1'	H15A	4	9	8.0	9.2	0.3	A15	H1'	H45A	4	9	8.1	8.0	0.3
**CH3** 5	A3	H1'	H171	4	9	5.4	8.5	1.2	A15	H1'	H474	4	9	5.3	7.7	0.5
	A3	H1'	H17A	4	9	5.6	7.9	1.3	A15	H1'	H47A	4	9	5.4	8.2	0.7
	A3	H1'	H17B	4	9	7.0	8.4	1.6	A15	H1'	H47B	4	9	6.8	8.5	0.4
	A3	H1'	H181	4	9	6.9	9.6	0.5	A15	H1'	H484	4	9	6.9	5.3	0.4
	A3	H1'	H18A	4	9	7.7	9.6	0.5	A15	H1'	H48A	4	9	7.9	5.9	0.5
	A3	H1'	H18B	4	9	8.1	10.3	0.5	A15	H1'	H48B	4	9	8.1	5.2	0.4
**CH2** 2	A3	H2	H131	4	9	6.4	7.5	0.4	A15	H2	H434	4	9	5.9	8.6	0.5
	A3	H2	H13A	4	9	7.5	6.5	0.4	A15	H2	H43A	4	9	7.4	9.5	0.5
**CH2** 3	A3	H2	H141	4	9	6.6	6.8	0.3	A15	H2	H444	4	9	5.2	6.7	0.5
	A3	H2	H14A	4	9	8.2	7.6	0.5	A15	H2	H44A	4	9	5.9	8.6	0.5
**CH2** 4	A3	H2	H151	4	9	6.2	9.6	0.4	A15	H2	H454	4	9	7.6	9.5	0.5
	A3	H2	H15A	4	9	7.9	9.2	0.4	A15	H2	H45A	4	9	8.0	8.2	0.7
**CH3** 5	A3	H2	H171	4	9	6.5	9.7	0.9	A15	H2	H474	4	9	6.0	9.3	0.7
	A3	H2	H17A	4	9	7.6	8.9	1.1	A15	H2	H47A	4	9	7.3	9.9	0.9
	A3	H2	H17B	4	9	8.2	9.3	1.4	A15	H2	H47B	4	9	7.8	9.8	0.6
	A3	H2	H181	4	9	8.5	10.6	0.5	A15	H2	H484	4	9	8.8	6.3	0.5
	A3	H2	H18A	4	9	8.6	10.9	0.4	A15	H2	H48A	4	9	8.9	7.0	0.6
	A3	H2	H18B	4	9	9.2	11.1	0.4	A15	H2	H48B	4	9	9.3	7.0	0.8
**CH2** 2	G4	H1'	H131	4	9	7.2	7.6	0.3	G16	H1'	H434	4	9	7.9	10.0	0.4
	G4	H1'	H13A	4	9	8.7	7.3	0.3	G16	H1'	H43A	4	9	9.0	10.0	0.4

**CH2** 3	G4	H1'	H141	4	9	7.8	7.4	0.4	G16	H1'	H444	4	9	6.0	7.7	0.5
	G4	H1'	H14A	4	9	9.1	8.8	0.4	G16	H1'	H44A	4	9	7.4	9.1	0.5
**CH2** 4	G4	H1'	H151	4	9	6.3	10.2	0.4	G16	H1'	H454	4	9	7.2	8.8	0.4
	G4	H1'	H15A	4	9	7.9	9.2	0.7	G16	H1'	H45A	4	9	8.4	7.4	0.4
**CH3** 5	G4	H1'	H171	4	9	5.9	10.4	0.9	G16	H1'	H474	4	9	4.7	7.0	0.5
	G4	H1'	H17A	4	9	7.3	9.7	1.0	G16	H1'	H47A	4	9	5.5	7.8	0.7
	G4	H1'	H17B	4	9	7.6	10.1	1.0	G16	H1'	H47B	4	9	6.0	7.6	1.0
	G4	H1'	H181	4	9	9.0	10.3	0.7	G16	H1'	H484	4	9	7.9	5.7	0.5
	G4	H1'	H18A	4	9	9.2	11.0	0.5	G16	H1'	H48A	4	9	8.3	5.2	0.5
	G4	H1'	H18B	4	9	9.3	11.2	0.5	G16	H1'	H48B	4	9	8.8	5.5	0.6

Table S3. Changes in ^{1}H (ΔH) and ^{13}C (ΔC) chemical shifts of the ligand DB213 upon passing from water to the RNA-bound state (ppm) in comparison with experiment. The labels of the atoms are reported in Chart 1 (main text). 2a, 2b refer to the two diastereotopic protons of this CH2 group. The values are averaged (<Δ>) over 250 snapshots during the QM/MM dynamics. Standard deviations (σ) are also reported.

		<Δ>	σ	Experiment
ΔH	**1** (CH arom)	0.03	0.33	0.00
	**2a** (CH2)	0.15	0.69	-0.12
	**2b** (CH2)	-0.03	0.66	-0.18
	**3** (CH2)	0.12	0.55	-0.02
	**4** (CH2)	0.06	0.54	0.01
	**5** (CH3)	0.00	0.39	0.03
ΔC	**1** (CH arom)	0.11	3.57	0.06
	**2** (CH2)	-1.33	4.81	0.06
	**3** (CH2)	-0.90	4.25	0.09
	**4** (CH2)	-0.08	6.53	-0.12
	**5** (CH3)	-0.49	3.92	0.02

Table S4. Selected distances (d in **Figure S9a**) in the QM/MM MD and in the 5 NMR structures. Here, the averaged values are along with the standard deviation (in brackets) for the QM/MM structure.

CAG RNA	DB213	QM/MM Average distance (Å)	NMR 1 distance (Å)	NMR 2 distance (Å)	NMR 3 distance (Å)	NMR 4 distance (Å)	NMR 5 distance (Å)
A15-OP2	N4-H29	1.7 (0.09)	not present	not present	not present	not present	not present
G4-O6	N5-H5	1.8 (0.09)	not present	not present	1.8	1.7	not present
G16-O6	N5-H7	1.8 (0.09)	not present	not present	1.7	not present	1.8
G16-O6	N5-H5	not present	not present	1.8	not present	not present	not present
A15-N7	N4-H29	not present	1.9	1.8	1.9	1.8	1.9
A3-N7	N3-H13	not present	1.8	1.8	1.8	1.9	1.8

Table S5. Angles α for the interactions of **Figure S9a**. Here, the averaged values are along with the standard deviation (in brackets) for the QM/MM structure.

CAG RNA	DB213	QM/MM Average angle (°)	NMR 1 Angle (°)	NMR 2 Angle (°)	NMR 3 Angle (°)	NMR 4 Angle (°)	NMR 5 Angle (°)
A15- OP2	N4-H29	163 (9)	not present	not present	not present	not present	not present
G4-O6	N5-H5	159 (9)	not present	not present	154	171	not present
G16-O6	N5-H7	155 (9)	not present	not present	163	not present	170
G16-O6	N5-H5	not present	not present	169	not present	not present	not present
A15-N7	N4-H29	not present	160	170	165	178	159
A3-N7	N3-H13	not present	179	172	171	170	175

Table S6. Angles θ for the interactions of **Figure S9b**.

CAG RNA	DB213	QM/MM Average angle (degree)	NMR 1 Angle (degree)	NMR 2 Angle (degree)	NMR 3 Angle (degree)	NMR 4 Angle (degree)	NMR 5 Angle (degree)
A15-N7	N4-H29	not present	48	44	50	54	56
A3-N7	N3-H13	not present	57	57	49	58	62

Table S7. Average distances and time courses for hydrogen bonds and salt bridges during classical MD simulation.

Type of interactions Present in the MD calculations	CAG RNA	DB213	Average distance (Å)	Time course (%)
H- bond	G4-O6	N5-H5	1.9 (0.08)	44.0%
H- bond	G16-O6	N5-H7	1.9 (0.08)	66.0%
H-bonded salt bridge	A15- OP2	N4-H29	1.9 (0.28)	100.0%

Table S8. Same as Table S2 except that we consider here the 100 ps classical MD simulation.

	RNA		DB213	restr		NMR	SIM	STD	RNA		DB213	restr		NMR	SIM	STD
CH2 2	C2	H1'	H131	4	9	8.5	10.8	0.6	C14	H1'	H434	4	9	9.4	10.0	0.6
	C2	H1'	H13A	4	9	9.0	10.7	0.7	C14	H1'	H43A	4	9	9.5	10.3	0.5
CH2 3	C2	H1'	H141	4	9	6.6	8.9	0.7	C14	H1'	H444	4	9	7.3	8.2	0.5
	C2	H1'	H14A	4	9	7.4	9.4	1.0	C14	H1'	H44A	4	9	8.2	7.8	0.4
CH2 4	C2	H1'	H151	4	9	8.3	10.1	1.1	C14	H1'	H454	4	9	8.5	9.7	0.4
	C2	H1'	H15A	4	9	8.7	9.8	0.7	C14	H1'	H45A	4	9	9.4	10.2	0.3
CH3 5	C2	H1'	H171	4	9	5.8	8.0	1.5	C14	H1'	H474	4	9	7.0	9.8	0.6
	C2	H1'	H17A	4	9	5.9	8.0	1.4	C14	H1'	H47A	4	9	7.4	10.1	0.6
	C2	H1'	H17B	4	9	7.3	8.5	2.2	C14	H1'	H47B	4	9	8.6	10.5	0.5
	C2	H1'	H181	4	9	7.4	9.2	1.2	C14	H1'	H484	4	9	7.7	8.8	1.0
	C2	H1'	H18A	4	9	8.3	8.3	1.7	C14	H1'	H48A	4	9	8.2	9.3	0.9
	C2	H1'	H18B	4	9	8.6	9.5	1.7	C14	H1'	H48B	4	9	9.1	9.1	0.7
CH2 2	A3	H8	H131	3	7	6.7	7.6	0.7	A15	H8	H434	3	7	6.7	5.6	0.5
	A3	H8	H13A	3	7	7.1	7.8	0.9	A15	H8	H43A	3	7	6.7	5.4	0.6
CH2 3	A3	H8	H141	3	7	4.8	6.2	1.1	A15	H8	H444	3	7	4.4	3.3	0.6
	A3	H8	H14A	3	7	5.8	7.3	1.4	A15	H8	H44A	3	7	4.6	3.5	0.5
CH2 4	A3	H8	H151	3	7	4.9	8.2	1.0	A15	H8	H454	3	7	5.2	5.5	0.4
	A3	H8	H15A	3	7	5.6	7.2	0.6	A15	H8	H45A	3	7	5.4	5.5	0.4
CH3 5	A3	H8	H171	3	7	3.3	7.2	1.9	A15	H8	H474	3	7	3.3	6.3	0.6
	A3	H8	H17A	3	7	4.2	7.3	1.5	A15	H8	H47A	3	7	4.3	6.5	0.5
	A3	H8	H17B	3	7	4.8	7.7	2.4	A15	H8	H47B	3	7	4.8	6.7	0.4
	A3	H8	H181	3	7	3.1	7.4	0.9	A15	H8	H484	3	7	3.0	4.5	1.1
	A3	H8	H18A	3	7	3.8	7.4	1.5	A15	H8	H48A	3	7	3.7	4.9	1.1
	A3	H8	H18B	3	7	4.6	8.4	1.3	A15	H8	H48B	3	7	4.5	5.0	0.7
CH2 2	A3	H1'	H131	4	9	8.3	10.8	0.7	A15	H1'	H434	4	9	8.1	8.6	0.3
	A3	H1'	H13A	4	9	9.3	10.5	0.9	A15	H1'	H43A	4	9	8.9	7.9	0.6
CH2 3	A3	H1'	H141	4	9	7.3	9.3	1.0	A15	H1'	H444	4	9	6.1	6.2	0.4
	A3	H1'	H14A	4	9	8.7	10.1	1.4	A15	H1'	H44A	4	9	6.7	7.0	0.5
CH2 4	A3	H1'	H151	4	9	6.8	11.4	1.0	A15	H1'	H454	4	9	7.8	8.9	0.3
	A3	H1'	H15A	4	9	8.0	10.7	0.6	A15	H1'	H45A	4	9	8.1	8.4	0.5
CH3 5	A3	H1'	H171	4	9	5.4	10.1	2.1	A15	H1'	H474	4	9	5.3	9.5	0.7
	A3	H1'	H17A	4	9	5.6	10.0	1.7	A15	H1'	H47A	4	9	5.4	9.9	0.5
	A3	H1'	H17B	4	9	7.0	10.6	2.5	A15	H1'	H47B	4	9	6.8	9.7	0.5
	A3	H1'	H181	4	9	6.9	11.0	1.0	A15	H1'	H484	4	9	6.9	7.0	1.1
	A3	H1'	H18A	4	9	7.7	10.7	1.6	A15	H1'	H48A	4	9	7.9	7.4	1.2
	A3	H1'	H18B	4	9	8.1	11.7	1.4	A15	H1'	H48B	4	9	8.1	7.4	0.7
CH2 2	A3	H2	H131	4	9	6.4	10.4	0.9	A15	H2	H434	4	9	5.9	8.7	0.6
	A3	H2	H13A	4	9	7.5	9.5	0.9	A15	H2	H43A	4	9	7.4	8.0	0.8
	A3	H2	H141	4	9	6.6	9.0	0.9	A15	H2	H444	4	9	5.2	7.5	0.8

CH2 3	A3	H2	H14A	4	9	8.2	9.2	1.3	A15	H2	H44A	4	9	5.9	8.1	0.7
CH2 4	A3	H2	H151	4	9	6.2	10.9	1.0	A15	H2	H454	4	9	7.6	10.2	0.6
	A3	H2	H15A	4	9	7.9	10.7	0.6	A15	H2	H45A	4	9	8.0	9.7	0.8
CH3 5	A3	H2	H171	4	9	6.5	9.8	2.2	A15	H2	H474	4	9	6.0	119	0.7
	A3	H2	H17A	4	9	7.6	9.3	1.8	A15	H2	H47A	4	9	7.3	12.0	0.7
	A3	H2	H17B	4	9	8.2	10.2	2.3	A15	H2	H47B	4	9	7.8	11.8	0.9
	A3	H2	H181	4	9	8.5	11.6	0.9	A15	H2	H484	4	9	8.8	9.5	1.3
	A3	H2	H18A	4	9	8.6	11.3	1.5	A15	H2	H48A	4	9	8.9	9.7	1.6
	A3	H2	H18B	4	9	9.2	11.8	1.4	A15	H2	H48B	4	9	9.3	9.9	0.9
CH2 2	G4	H1'	H131	4	9	7.2	8.4	0.8	G16	H1'	H434	4	9	7.9	9.6	0.6
	G4	H1'	H13A	4	9	8.7	7.8	0.9	G16	H1'	H43A	4	9	9.0	8.3	0.9
CH2 3	G4	H1'	H141	4	9	7.8	7.5	1.1	G16	H1'	H444	4	9	6.0	7.6	0.6
	G4	H1'	H14A	4	9	9.1	8.2	1.5	G16	H1'	H44A	4	9	7.4	9.0	0.6
CH2 4	G4	H1'	H151	4	9	6.3	9.8	0.9	G16	H1'	H454	4	9	7.2	10.2	0.7
	G4	H1'	H15A	4	9	7.9	9.2	0.5	G16	H1'	H45A	4	9	8.4	9.0	0.9
CH3 5	G4	H1'	H171	4	9	5.9	9.5	2.4	G16	H1'	H474	4	9	4.7	10.9	1.1
	G4	H1'	H17A	4	9	7.3	9.2	1.7	G16	H1'	H47A	4	9	5.5	11.3	0.7
	G4	H1'	H17B	4	9	7.6	9.9	2.2	G16	H1'	H47B	4	9	6.0	10.7	1.2
	G4	H1'	H181	4	9	9.0	10.3	0.8	G16	H1'	H484	4	9	7.9	8.0	1.3
	G4	H1'	H18A	4	9	9.2	10.5	1.3	G16	H1'	H48A	4	9	8.3	8.2	1.4
	G4	H1'	H18B	4	9	9.3	10.9	1.1	G16	H1'	H48B	4	9	8.8	8.3	0.9

Table S9. Same as Table S3 except that we consider here the classical MD simulation.

		<Δ>	σ	Experiment
ΔH	**1** (CH arom)	0.01	0.25	0.00
	**2a** (CH2)	0.21	0.42	-0.12
	**2b** (CH2)	-0.26	0.44	-0.18
	**3** (CH2)	-0.03	0.22	-0.02
	**4** (CH2)	-0.15	0.25	0.01
	**5** (CH3)	-0.03	0.13	0.03
ΔC	**1** (CH arom)	-1.04	4.12	0.06
	**2** (CH2)	-0.65	5.71	0.06
	**3** (CH2)	-0.77	3.71	0.09
	**4** (CH2)	-0.63	5.32	-0.12
	**5** (CH3)	-0.10	3.29	0.02

REFERENCES

(1) G, H. On Chorea. *Medical and Surgical Reporter: A Weekly Journal* **1872**, *26*, 5.
(2) Lieberman, A. P.; Shakkottai, V. G.; Albin, R. L. Polyglutamine Repeats in Neurodegenerative Diseases. *Annu Rev Pathol* **2019**, *14*, 1-27. DOI: 10.1146/annurev-pathmechdis-012418-012857 From NLM Medline.
(3) Depienne, C.; Mandel, J. L. 30 years of repeat expansion disorders: What have we learned and what are the remaining challenges? *Am J Hum Genet* **2021**, *108* (5), 764-785. DOI: 10.1016/j.ajhg.2021.03.011 From NLM Medline.
(4) A novel gene containing a trinucleotide repeat that is expanded and unstable on Huntington's disease chromosomes. The Huntington's Disease Collaborative Research Group. *Cell* **1993**, *72* (6), 971-983. DOI: 10.1016/0092-8674(93)90585-e From NLM Medline.
(5) Myers, R. H. Huntington's disease genetics. *NeuroRx* **2004**, *1* (2), 255-262. DOI: 10.1602/neurorx.1.2.255 From NLM Medline.
(6) Walker, F. O. Huntington's disease. *The Lancet* **2007**, *369* (9557), 218-228.
(7) Stoyas, C. A.; La Spada, A. R. The CAG-polyglutamine repeat diseases: a clinical, molecular, genetic, and pathophysiologic nosology. *Handb Clin Neurol* **2018**, *147*, 143-170. DOI: 10.1016/B978-0-444-63233-3.00011-7 From NLM Medline.
(8) DiFiglia, M.; Sapp, E.; Chase, K. O.; Davies, S. W.; Bates, G. P.; Vonsattel, J. P.; Aronin, N. Aggregation of huntingtin in neuronal intranuclear inclusions and dystrophic neurites in brain. *Science* **1997**, *277* (5334), 1990-1993. DOI: 10.1126/science.277.5334.1990 From NLM Medline.
(9) Broda, M.; Kierzek, E.; Gdaniec, Z.; Kulinski, T.; Kierzek, R. Thermodynamic stability of RNA structures formed by CNG trinucleotide repeats. Implication for prediction of RNA structure. *Biochemistry* **2005**, *44* (32), 10873-10882. DOI: 10.1021/bi0502339 From NLM Medline.
(10) Kiliszek, A.; Kierzek, R.; Krzyzosiak, W. J.; Rypniewski, W. Atomic resolution structure of CAG RNA repeats: structural insights and implications for the trinucleotide repeat expansion diseases. *Nucleic Acids Res* **2010**, *38* (22), 8370-8376. DOI: 10.1093/nar/gkq700 From NLM Medline.
(11) Michlewski, G.; Krzyzosiak, W. J. Molecular architecture of CAG repeats in human disease related transcripts. *J Mol Biol* **2004**, *340* (4), 665-679. DOI: 10.1016/j.jmb.2004.05.021 From NLM Medline.
(12) Sobczak, K.; de Mezer, M.; Michlewski, G.; Krol, J.; Krzyzosiak, W. J. RNA structure of trinucleotide repeats associated with human neurological diseases. *Nucleic Acids Res* **2003**, *31* (19), 5469-5482. DOI: 10.1093/nar/gkg766 From NLM Medline.
(13) Krauss, S.; Griesche, N.; Jastrzebska, E.; Chen, C.; Rutschow, D.; Achmuller, C.; Dorn, S.; Boesch, S. M.; Lalowski, M.; Wanker, E.; et al. Translation of HTT mRNA with expanded CAG repeats is regulated by the MID1-PP2A protein complex. *Nat Commun* **2013**, *4*, 1511. DOI: 10.1038/ncomms2514 From NLM Medline.
(14) Heinz, A.; Nabariya, D. K.; Krauss, S. Huntingtin and Its Role in Mechanisms of RNA-Mediated Toxicity. *Toxins (Basel)* **2021**, *13* (7). DOI: 10.3390/toxins13070487 From NLM Medline.
(15) Nalavade, R.; Griesche, N.; Ryan, D. P.; Hildebrand, S.; Krauss, S. Mechanisms of RNA-induced toxicity in CAG repeat disorders. *Cell Death Dis* **2013**, *4* (8), e752. DOI: 10.1038/cddis.2013.276 From NLM Medline.
(16) Neueder, A. RNA-Mediated Disease Mechanisms in Neurodegenerative Disorders. *J Mol Biol* **2019**, *431* (9), 1780-1791. DOI: 10.1016/j.jmb.2018.12.012 From NLM Medline.

(17) Schilling, J.; Broemer, M.; Atanassov, I.; Duernberger, Y.; Vorberg, I.; Dieterich, C.; Dagane, A.; Dittmar, G.; Wanker, E.; van Roon-Mom, W.; et al. Deregulated Splicing Is a Major Mechanism of RNA-Induced Toxicity in Huntington's Disease. *J Mol Biol* **2019**, *431* (9), 1869-1877. DOI: 10.1016/j.jmb.2019.01.034 From NLM Medline.
(18) Banez-Coronel, M.; Porta, S.; Kagerbauer, B.; Mateu-Huertas, E.; Pantano, L.; Ferrer, I.; Guzman, M.; Estivill, X.; Marti, E. A pathogenic mechanism in Huntington's disease involves small CAG-repeated RNAs with neurotoxic activity. *PLoS Genet* **2012**, *8* (2), e1002481. DOI: 10.1371/journal.pgen.1002481 From NLM Medline.
(19) Li, L. B.; Yu, Z.; Teng, X.; Bonini, N. M. RNA toxicity is a component of ataxin-3 degeneration in Drosophila. *Nature* **2008**, *453* (7198), 1107-1111. DOI: 10.1038/nature06909 From NLM Medline.
(20) Sun, X.; Li, P. P.; Zhu, S.; Cohen, R.; Marque, L. O.; Ross, C. A.; Pulst, S. M.; Chan, H. Y.; Margolis, R. L.; Rudnicki, D. D. Nuclear retention of full-length HTT RNA is mediated by splicing factors MBNL1 and U2AF65. *Sci Rep* **2015**, *5*, 12521. DOI: 10.1038/srep12521 From NLM Medline.
(21) Baltrukevich, H.; Bartos, P. RNA-protein complexes and force field polarizability. *Front Chem* **2023**, *11*, 1217506. DOI: 10.3389/fchem.2023.1217506 From NLM PubMed-not-MEDLINE.
(22) Harger, M.; Li, D.; Wang, Z.; Dalby, K.; Lagardere, L.; Piquemal, J. P.; Ponder, J.; Ren, P. Tinker-OpenMM: Absolute and relative alchemical free energies using AMOEBA on GPUs. *J Comput Chem* **2017**, *38* (23), 2047-2055. DOI: 10.1002/jcc.24853 From NLM Medline.
(23) Zhang, C.; Lu, C.; Jing, Z.; Wu, C.; Piquemal, J. P.; Ponder, J. W.; Ren, P. AMOEBA Polarizable Atomic Multipole Force Field for Nucleic Acids. *J Chem Theory Comput* **2018**, *14* (4), 2084-2108. DOI: 10.1021/acs.jctc.7b01169 From NLM Medline.
(24) Yang, X.; Liu, C.; Ren, P. Exploring Biomolecular Conformational Dynamics with Polarizable Force Field AMOEBA and Enhanced Sampling Method Milestoning. *J Chem Theory Comput* **2024**, *20* (10), 4065-4075. DOI: 10.1021/acs.jctc.4c00053 From NLM Medline.
(25) Peng, S.; Guo, P.; Lin, X.; An, Y.; Sze, K. H.; Lau, M. H. Y.; Chen, Z. S.; Wang, Q.; Li, W.; Sun, J. K.; et al. CAG RNAs induce DNA damage and apoptosis by silencing NUDT16 expression in polyglutamine degeneration. *Proc Natl Acad Sci U S A* **2021**, *118* (19). DOI: 10.1073/pnas.2022940118 From NLM Medline.
(26) Perez, A.; Marchan, I.; Svozil, D.; Sponer, J.; Cheatham, T. E., 3rd; Laughton, C. A.; Orozco, M. Refinement of the AMBER force field for nucleic acids: improving the description of alpha/gamma conformers. *Biophys J* **2007**, *92* (11), 3817-3829. DOI: 10.1529/biophysj.106.097782 From NLM Medline.
(27) Joung, I. S.; Cheatham, T. E., 3rd. Determination of alkali and halide monovalent ion parameters for use in explicitly solvated biomolecular simulations. *J Phys Chem B* **2008**, *112* (30), 9020-9041. DOI: 10.1021/jp8001614 From NLM Medline.
(28) Jorgensen, W. L.; Chandrasekhar, J.; Madura, J. D.; Impey, R. W.; Klein, M. L. Comparison of simple potential functions for simulating liquid water. *The Journal of chemical physics* **1983**, *79* (2), 926-935.
(29) Wang, J.; Cieplak, P.; Kollman, P. A. How well does a restrained electrostatic potential (RESP) model perform in calculating conformational energies of organic and biological molecules? *Journal of computational chemistry* **2000**, *21* (12), 1049-1074.
(30) M. J. Frisch, G. W. T., H. B. Schlegel, G. E. Scuseria, M. A. Robb, J. R. Cheeseman, G. Scalmani, V. Barone, G. A. Petersson, H. Nakatsuji, X. Li, M. Caricato, A. Marenich, J. Bloino, B. G. Janesko, R. Gomperts, B. Mennucci, H. P. Hratchian, J. V. Ortiz, A. F. Izmaylov, J. L. Sonnenberg, D. Williams-Young, F. Ding, F. Lipparini, F. Egidi, J. Goings, B. Peng, A. Petrone, T. Henderson, D. Ranasinghe, V. G. Zakrzewski, J. Gao, N. Rega, G. Zheng, W. Liang, M. Hada, M. Ehara, K. Toyota,

R. Fukuda, J. Hasegawa, M. Ishida, T. Nakajima, Y. Honda, O. Kitao, H. Nakai, T. Vreven, K. Throssell, J. A. Montgomery, Jr., J. E. Peralta, F. Ogliaro, M. Bearpark, J. J. Heyd, E. Brothers, K. N. Kudin, V. N. Staroverov, T. Keith, R. Kobayashi, J. Normand, K. Raghavachari, A. Rendell, J. C. Burant, S. S. Iyengar, J. Tomasi, M. Cossi, J. M. Millam, M. Klene, C. Adamo, R. Cammi, J. W. Ochterski, R. L. Martin, K. Morokuma, O. Farkas, J. B. Foresman, and D. J. Fox, Gaussian, Inc., Wallingford CT. Gaussian 09, Revision A.02. **2016**.

(31) Wang, J.; Wolf, R. M.; Caldwell, J. W.; Kollman, P. A.; Case, D. A. Development and testing of a general amber force field. *J Comput Chem* **2004**, *25* (9), 1157-1174. DOI: 10.1002/jcc.20035 From NLM Medline.

(32) Hess, B.; Bekker, H.; Berendsen, H. J.; Fraaije, J. G. LINCS: a linear constraint solver for molecular simulations. *Journal of computational chemistry* **1997**, *18* (12), 1463-1472.

(33) Darden, T.; York, D.; Pedersen, L. Particle mesh Ewald: An N· log (N) method for Ewald sums in large systems. *The Journal of chemical physics* **1993**, *98* (12), 10089-10092.

(34) Essmann, U.; Perera, L.; Berkowitz, M. L.; Darden, T.; Lee, H.; Pedersen, L. G. A smooth particle mesh Ewald method. *The Journal of chemical physics* **1995**, *103* (19), 8577-8593.

(35) Evans, D. J.; Holian, B. L. The nose–hoover thermostat. *The Journal of chemical physics* **1985**, *83* (8), 4069-4074.

(36) Parrinello, M.; Rahman, A. Polymorphic transitions in single crystals: A new molecular dynamics method. *Journal of Applied physics* **1981**, *52* (12), 7182-7190.

(37) Bussi, G.; Donadio, D.; Parrinello, M. Canonical sampling through velocity rescaling. *J Chem Phys* **2007**, *126* (1), 014101. DOI: 10.1063/1.2408420 From NLM PubMed-not-MEDLINE.

(38) Berendsen, H. J.; Postma, J. v.; Van Gunsteren, W. F.; DiNola, A.; Haak, J. R. Molecular dynamics with coupling to an external bath. *The Journal of chemical physics* **1984**, *81* (8), 3684-3690.

(39) Haug, E.; Arora, J.; Matsui, K. A steepest-descent method for optimization of mechanical systems. *Journal of Optimization Theory and Applications* **1976**, *19*, 401-424.

(40) Hestenes, M. R.; Stiefel, E. *Methods of conjugate gradients for solving linear systems*; NBS Washington, DC, 1952.

(41) Becke, A. D. Density-functional exchange-energy approximation with correct asymptotic behavior. *Phys Rev A Gen Phys* **1988**, *38* (6), 3098-3100. DOI: 10.1103/physreva.38.3098 From NLM PubMed-not-MEDLINE.

(42) Lee, C.; Yang, W.; Parr, R. G. Development of the Colle-Salvetti correlation-energy formula into a functional of the electron density. *Phys Rev B Condens Matter* **1988**, *37* (2), 785-789. DOI: 10.1103/physrevb.37.785 From NLM PubMed-not-MEDLINE.

(43) CPMD, http://www.cpmd.org/, Copyright IBM Corp 1990-2023, Copyright MPI für Festkörperforschung Stuttgart 1997-2001 (accessed.

(44) Troullier, N.; Martins, J. L. Efficient pseudopotentials for plane-wave calculations. *Phys Rev B Condens Matter* **1991**, *43* (3), 1993-2006. DOI: 10.1103/physrevb.43.1993 From NLM PubMed-not-MEDLINE.

(45) Martyna, G. J.; Tuckerman, M. E. A reciprocal space based method for treating long range interactions in ab initio and force-field-based calculations in clusters. *The Journal of chemical physics* **1999**, *110* (6), 2810-2821.

(46) Olsen, J. M. H.; Bolnykh, V.; Meloni, S.; Ippoliti, E.; Bircher, M. P.; Carloni, P.; Rothlisberger, U. MiMiC: A Novel Framework for Multiscale Modeling in Computational Chemistry. *J Chem Theory Comput* **2019**, *15* (6), 3810-3823. DOI: 10.1021/acs.jctc.9b00093 From NLM PubMed-not-MEDLINE.

(47) Laio, A.; VandeVondele, J.; Rothlisberger, U. A Hamiltonian electrostatic coupling scheme for hybrid Car–Parrinello molecular dynamics simulations. *The Journal of chemical physics* **2002**, *116* (16), 6941-6947.
(48) Born, M.; Oppenheimer, R. On the quantum theory of molecules. In *Quantum Chemistry: Classic Scientific Papers*, World Scientific, 2000; pp 1-24.
(49) Nosé, S. A unified formulation of the constant temperature molecular dynamics methods. *The Journal of chemical physics* **1984**, *81* (1), 511-519.
(50) Hoover, W. G. Canonical dynamics: Equilibrium phase-space distributions. *Physical review A* **1985**, *31* (3), 1695.
(51) Martyna, G. J.; Klein, M. L.; Tuckerman, M. Nosé–Hoover chains: The canonical ensemble via continuous dynamics. *The Journal of chemical physics* **1992**, *97* (4), 2635-2643.
(52) Raghavan, B.; Schackert, F. K.; Levy, A.; Johnson, S. K.; Ippoliti, E.; Mandelli, D.; Olsen, J. M. H.; Rothlisberger, U.; Carloni, P. MiMiCPy: An Efficient Toolkit for MiMiC-Based QM/MM Simulations. *J Chem Inf Model* **2023**, *63* (5), 1406-1412. DOI: 10.1021/acs.jcim.2c01620 From NLM Medline.
(53) Gao, J.; Xia, X. A priori evaluation of aqueous polarization effects through Monte Carlo QM-MM simulations. *Science* **1992**, *258* (5082), 631-635.
(54) Capelli, R.; Lyu, W.; Bolnykh, V.; Meloni, S.; Olsen, J. M. H.; Rothlisberger, U.; Parrinello, M.; Carloni, P. Accuracy of Molecular Simulation-Based Predictions of k(off) Values: A Metadynamics Study. *J Phys Chem Lett* **2020**, *11* (15), 6373-6381. DOI: 10.1021/acs.jpclett.0c00999 From NLM Medline.
(55) Carloni, P.; Andreoni, W.; Hutter, J.; Curioni, A.; Giannozzi, P.; Parrinello, M. Structure and bonding in cisplatin and other Pt (II) complexes. *Chemical physics letters* **1995**, *234* (1-3), 50-56.
(56) *cube-tools*. https://pypi.org/project/cpmd-cube-tools/ (accessed).
(57) Neese, F.; Wennmohs, F.; Becker, U.; Riplinger, C. The ORCA quantum chemistry program package. *J Chem Phys* **2020**, *152* (22), 224108. DOI: 10.1063/5.0004608 From NLM PubMed-not-MEDLINE.
(58) *ORCA*. https://orcaforum.kofo.mpg.de/app.php/portal (accessed).
(59) Poidevin, C.; Stoychev, G. L.; Riplinger, C.; Auer, A. A. High Level Electronic Structure Calculation of Molecular Solid-State NMR Shielding Constants. *J Chem Theory Comput* **2022**, *18* (4), 2408-2417. DOI: 10.1021/acs.jctc.1c01095 From NLM Medline.
(60) Rock, M.; Heel, S. V.; Juen, F. S.; Eidelpes, R.; Kreutz, C.; Breuker, K.; Tollinger, M. The PR-10 Protein Pru p 1 is an Endonuclease that Preferentially Cleaves Single-Stranded RNA. *Chembiochem* **2024**, *25* (12), e202400204. DOI: 10.1002/cbic.202400204 From NLM Medline.
(61) Marcheschi, R. J.; Tonelli, M.; Kumar, A.; Butcher, S. E. Structure of the HIV-1 frameshift site RNA bound to a small molecule inhibitor of viral replication. *ACS Chem Biol* **2011**, *6* (8), 857-864. DOI: 10.1021/cb200082d From NLM Medline.
(62) Hwang, T.-L.; Shaka, A. Water suppression that works. Excitation sculpting using arbitrary wave-forms and pulsed-field gradients. *Journal of Magnetic Resonance, Series A* **1995**, *112* (2), 275-279.
(63) Bissantz, C.; Kuhn, B.; Stahl, M. A medicinal chemist's guide to molecular interactions. *J Med Chem* **2010**, *53* (14), 5061-5084. DOI: 10.1021/jm100112j From NLM Medline.
(64) Nobeli, I.; Price, S.; Lommerse, J.; Taylor, R. Hydrogen bonding properties of oxygen and nitrogen acceptors in aromatic heterocycles. *Journal of Computational Chemistry* **1997**, *18* (16), 2060-2074.
(65) Berdnikova, D. V.; Carloni, P.; Krauss, S.; Rossetti, G. Role and Perspective of Molecular Simulation-Based Investigation of RNA-Ligand Interaction: From Small Molecules and Peptides to

Photoswitchable RNA Binding. *Molecules* **2021**, *26* (11). DOI: 10.3390/molecules26113384 From NLM Medline.
